# Optogenetic dissection of ictal propagation in the hippocampal–entorhinal cortex structures

**DOI:** 10.1038/ncomms10962

**Published:** 2016-03-21

**Authors:** Yi Lu, Cheng Zhong, Lulu Wang, Pengfei Wei, Wei He, Kang Huang, Yi Zhang, Yang Zhan, Guoping Feng, Liping Wang

**Affiliations:** 1 Shenzhen Key Lab of Neuropsychiatric Modulation and Collaborative Innovation Center for Brain Science, CAS Center for Excellence in Brain Science and Intelligence Technology, the Brain Cognition and Brain Disease Institute (BCBDI) for Collaboration Research of SIAT at CAS and the McGovern Institute at MIT, Shenzhen Institutes of Advanced Technology, Chinese Academy of Sciences, Shenzhen 518055, China; 2 University of Chinese Academy of Sciences, Beijing 100049, China; 3 McGovern Institute for Brain Research, Department of Brain and Cognitive Sciences, MIT, Cambridge, Massachusetts 02139, USA

## Abstract

Temporal lobe epilepsy (TLE) is one of the most common drug-resistant forms of epilepsy in adults and usually originates in the hippocampal formations. However, both the network mechanisms that support the seizure spread and the exact directions of ictal propagation remain largely unknown. Here we report the dissection of ictal propagation in the hippocampal–entorhinal cortex (HP–EC) structures using optogenetic methods in multiple brain regions of a kainic acid-induced model of TLE in VGAT-ChR2 transgenic mice. We perform highly temporally precise cross-area analyses of epileptic neuronal networks and find a feed-forward propagation pathway of ictal discharges from the dentate gyrus/hilus (DGH) to the medial entorhinal cortex, instead of a re-entrant loop. We also demonstrate that activating DGH GABAergic interneurons can significantly inhibit the spread of ictal seizures and largely rescue behavioural deficits in kainate-exposed animals. These findings may shed light on future therapeutic treatments of TLE.

## Abstract (web summary)

The network mechanism supporting seizure spread in temporal lobe epilepsy (TLE) is only partially understood. Using optogenetic methods, Lu *et al.* identify a feed-forward propagation pathway of ictal discharges from the dentate gyrus/hilus to the medial entorhinal cortex in a mouse model of TLE.

## Graphical abstract

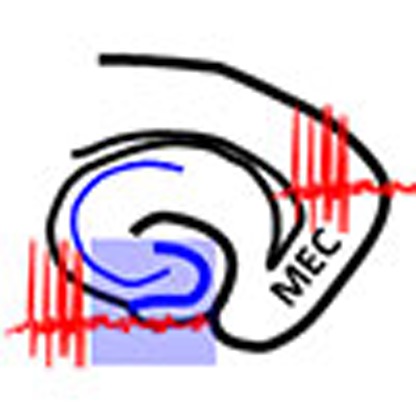

Temporal lobe epilepsy (TLE) is one of the most common types of epilepsy in adults. It is a circuit-level syndrome characterized by spontaneous seizures originating from the hippocampal and parahippocampal (HP–PHP) structures^1^,^2^,^3^. Until recently, relatively little has been known about the underlying mechanisms of ictal propagation in the pathological circuitry. Dissection of these mechanisms of propagation within the HP–PHP structures is crucially important for the development of specific therapeutic targets to more effectively treat TLE^2^,^4^.

During seizures, hypersynchronous discharges of interconnected excitatory neurons in the ictal core spread to the efferent brain regions and recruit neighbouring neurons into an abnormally synchronous pattern^5^. Many researchers have used classical patch-clamp analysis in hippocampal–entorhinal cortex (HP–EC) brain slices to identify the propagation path of ictal activity in the aberrant circuitry. These studies revealed that ictal discharges are initiated in the EC, propagate via the dentate gyrus (DG) to hippocampal regions CA3 and CA1, and subsequently re-enter the EC (EC-DG-CA3-CA1-EC re-entrant loop)^6^,^7^,^8^,^9^. Other studies using *in vitro* isolated guinea pig brains demonstrated that ictal discharges might propagate via a DG-CA1-EC-DG re-entrant loop^3^,^10^. The exact subregion of the pathological circuitry that dominates ictal propagation remained largely unclear, however. Recent studies suggested that multi-site *in vivo* recording and manipulation would be critical for investigating the propagation pathway of ictal discharges, as it could provide highly precise spatiotemporal insight into the dynamics of seizures in different brain regions at the organism level^1^,^2^,^11^. Although data from simultaneously recorded local field potentials (LFPs) and electroencephalographs suggested that the earliest epileptic seizures most frequently originate in the hippocampal formation^2^,^11^, the spread of ictal discharges in the HP–EC structures had not yet been fully characterized *in vivo* due to certain limitations in current methodologies.

Epileptic seizures are generally recognized as a consequence of excitation/inhibition (E/I) imbalance in involved neuronal networks^12^. Restoring appropriate E/I balance by cell-specific modulation at proper sites in the epileptic circuitry is crucial for intervention in seizures. The function of inhibitory neurons has attracted increasing attention due to their essential role in the regulation of neuronal activity and network synchronization, as well as the coordination of animal behaviours^13^,^14^,^15^. Because classical electrophysiological techniques lack cell-type specificity, advances in optogenetics have only recently allowed us to characterize and manipulate the pathological circuitry underlying seizures *in vivo*, with high temporal precision and with particular regard to GABAergic interneurons (INs). Although optogenetic techniques have already shown significant efficacy in interrupting seizure activity in epileptic animals^4^,^16^,^17^,^18^,^19^, circuit-level dissection of ictal propagation in the HP–PHP structures has not been adequately conducted at millisecond timescales and under cell-specific conditions *in vivo*. As a result, the exact subregion of the HP–EC structure that dominates the propagation of ictal discharges is also still unknown.

To address circuit-level mechanisms controlling seizure activity, we develop novel neural probes for drug delivery as well as multi-site optical stimulation and electrophysiological recording *in vivo*. We induce TLE seizures by direct microinjection of kainic acid (KA) into the dorsal hippocampus of VGAT-ChR2 transgenic mice^20^, and obtain multi-channel recordings from multiple brain regions including dentate gyrus/hilus (DGH), medial entorhinal cortex (MEC) and primary motor cortex (M1). On the basis multi-region electrical recordings, we find significantly increased synchronizations between the recorded regions during ictal seizures. To investigate the temporal relationship between these regions, we analyse the cross-area spike-triggered average of the LFPs (stLFPs) and the distribution of the cross-correlation peak lags of the LFPs. Instead of a re-entrant propagating loop, our data directly suggest a feed-forward propagation pathway of ictal discharges from the DGH to MEC. We then use optogenetic manipulations of ChR2-expressing GABAergic INs to verify our finding. Selectively activating DGH GABAergic INs instantly suppress ictal propagation and rescue behavioural deficits in KA-treated mice, while exciting MEC GABAergic INs show no significant effect. These results confirm a feed-forward direction of ictal propagation from the DGH to MEC and demonstrate that hypersynchronous DGH neurons may dominate the ictal discharges. They also may shed new light on underlying network mechanisms of seizure suppression, suggesting DGH neurons as potential targets for therapeutic TLE interventions. Furthermore, as the hippocampal formation also plays an important role in learning, memory, spatial navigation, emotion and social behaviours, functional dissection of HP–EC circuitry may also contribute to understanding other neuropsychiatric disorders including Alzheimer's disease, and emotive and cognitive impairments^21^,^22^,^23^.

## Results

### Increased synchrony results in ictal propagation

**Figure 1:**
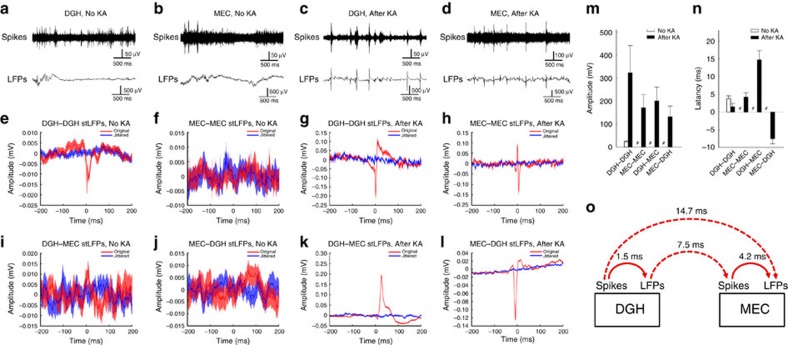
Spike-triggered average of LFP (stLFP) indicates the propagating direction of ictal seizures. (**a**–**d**) Raw spikes (upper panels) and LFPs (lower panels) recorded from representative channels in the DGH (**a**,**c**) and MEC (**b**,**d**) before (**a**,**b**) and after (**c**,**d**) kainate. (**e**–**h**) DGH spike-triggered averages of DGH LFPs (**e**,**g**: DGH–DGH stLFPs, *n*=5 mice) and MEC spike-triggered averages of MEC LFPs (**f**,**h**; MEC–MEC stLFPs, *n*=6 mice) before (**e**,**f**) and after (**g**,**h**) kainate. (**i**–**l**): Cross-area DGH spike-triggered averages of MEC LFPs (**i**,**k**; DGH–MEC stLFPs, *n*=5 mice) and MEC spike-triggered averages of DGH LFPs (**j**,**l**; MEC–DGH stLFPs, *n*=6 mice) before (**i**,**j**) and after (**k**,**l**) kainate. (**m**,**n**) Averaged peak aptitudes (**m**) and latencies (**n**) in stLFPs. Error bars represent s.d. #Represents data not available. (**o**) Proposed propagation direction of ictal seizures according to the stLFPs.

To investigate network dynamics in the pathological circuitry affected by TLE *in vivo*, custom-made neural probes were implanted into multiple brain regions simultaneously (Supplementary Fig. 1). KA was microinjected into the right dorsal hippocampus to induce an acute status epilepticus. Ictal discharges started ∼30 min after kainate administration and were characterized by significantly increased numbers of large-amplitude spikes and LFPs. During this period, multi-unit bursts frequently replaced single units in the DGH and MEC (Fig. 1a–d), suggesting hypersynchronization of the affected neurons^24^,^25^. We thus calculated stLFPs to quantify synchronization and study the temporal dynamics in the epileptic circuitry during ictal seizures^26^,^27^. In normal animals, only a weak peak appeared in DGH spike-triggered averages of DGH LFPs (DGH–DGH stLFPs, *n*=5 mice; Fig. 1e), probably due to the burst firing of DGH principal neurons (PNs; Supplementary Fig. 3f). No obvious peak in MEC spike-triggered averages of MEC LFPs was observed (MEC–MEC stLFPs, *n*=6 mice; Fig. 1f). After the onset of ictal seizures, strong DGH–DGH and MEC–MEC stLFP peaks emerged (Fig. 1g–h), with average amplitudes of 323.0±118.6 and 171.2±58.8 μV (mean±s.d.; Fig. 1m) and average latencies of 1.5±1.0 and 4.2±1.2 ms, respectively (mean±s.d.; Fig. 1n). This suggests that the LFP fluctuations were triggered by the hypersynchronous multi-unit activity of local neurons.

We next asked whether local neural activities also had a temporal relationship with LFPs in a distant region of the HP–EC circuitry. Before kainate administration, no apparent peak was observed in DGH spike-triggered averages of MEC LFPs (DGH–MEC stLFPs, *n*=5 mice; Fig. 1i) or in the MEC spike-triggered averages of DGH LFPs (MEC–DGH stLFPs, *n*=6 mice; Fig. 1j). However, in the ictal phase, strong peaks in both DGH–MEC and MEC–DGH stLFPs were observed (Fig. 1k–l), with average amplitudes of 201.4±60.2 and 132.3±46.4 μV, respectively (mean±s.d.; Fig. 1m), suggesting a strong temporal relationship between the DGH and MEC. Interestingly, we found that average peak latencies in the DGH–MEC and MEC–DGH stLFPs were 14.7±2.6 (mean±s.d.; Fig. 1n) and −7.5±1.4 ms (mean±s.d., Fig. 1n), respectively. The increase in absolute value of cross-area stLFP latency may be due to the prolonged travelling distance of ictal discharges. A positive or negative shift in the cross-area stLFP peak is associated with postsynaptic or presynaptic responses, respectively, representing the directionality in ictal propagation. The results therefore imply that MEC spikes were triggered by DGH LFPs and MEC LFPs by DGH spikes. Altogether, it suggests a propagation direction of DGH spikes→DGH LFPs→MEC spikes→MEC LFPs (Fig. 1o), indicating that ictal discharges in the MEC may be triggered by the activity of hypersynchronous DGH neurons.

### DGH PNs dominates ictal propagation

To corroborate the above findings, we used VGAT-ChR2(H134R)-EYFP bacterial artificial chromosome transgenic mice to test whether hyperexcitability of neurons in the HP–EC structures could be suppressed by the optogenetic activation of GABAergic INs (experimental design shown in Supplementary Fig. 2). Specific expression of ChR2 in GABAergic INs was evidenced by high co-localization of endogenous YFP and GAD67 in DGH and MEC horizontal sections (Supplementary Figs 3a and 4a)^28^. The function of ChR2-positive neurons was verified by electrophysiological analyses *in vivo* (Supplementary Figs 3b–h and 4b–h), which suggested that optical stimulation significantly activated GABAergic INs and suppressed the activity of local PNs.

**Figure 2:**
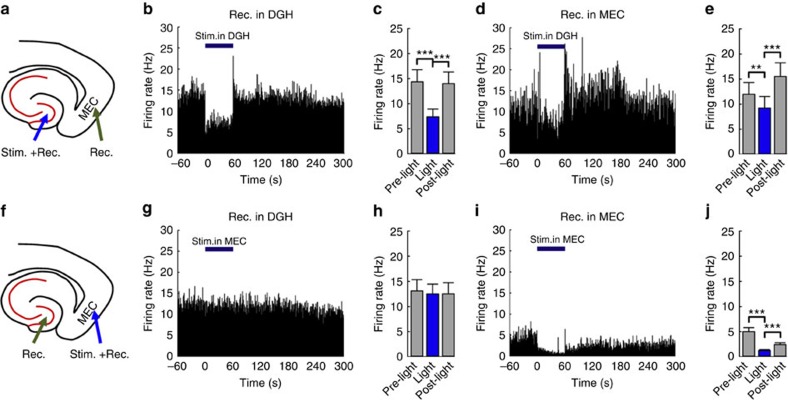
Selectively activating GABAergic interneurons inhibits multi-unit activity during ictal seizures. (**a**,**f**) Targeting sites for optical stimulation and electrical recording *in vivo*. (**b**,**d**,**g**,**i**) Representative examples of multi-unit firing rates in the DGH (**b**,**g**) and MEC (**d**,**i**) at the indicated times before, during and after 60 s optical stimulation. (**c**,**e**,**h**,**j**) Averaged multi-unit firing rates in the DGH (**c**, *n*=61 from 5 mice; **h**, *n*=47 from 5 mice) and MEC (**e**, *n*=48 from 5 mice; **j**, *n*=43 from 5 mice), respectively. Light pulses (473 nm, 5 ms pulse duration at 130 Hz) were delivered into the DGH (**a**–**e**) and MEC (**f**–**j**) at time 0. The thick blue line denotes the 60-s stimulation period. Error bars represent s.e.m. Stars represent significant differences (*P<0.05, **P<0.01, ***P<0.001, paired *t*-test.).

During ictal seizures, spontaneous multi-unit bursts were detected in the DGH (*n*=104 neurons from 10 mice) and MEC (*n*=95 neurons from 10 mice) (Fig. 2). An optimized stimulation frequency of 130 Hz was chosen and evaluated by its inhibitory efficiency on local ictal activity (Supplementary Fig. 5). Selectively activating DGH (*n*=61 from 5 mice; Fig. 2a–c) or MEC GABAergic INs (*n*=47 from 5 mice; Fig. 2f,i,j) significantly decreased the firing rates of local neurons. However, we found that activating DGH GABAergic INs also inhibited multi-unit firings in the MEC (*n*=48 from 5 mice; Fig. 2d,e), whereas activating MEC GABAergic INs showed no significant effect on the activity of DGH neurons (*n*=43 from 5 mice; Fig. 2g,h). This suggests that DGH ictal discharges may have a governing influence on the activity of MEC neurons, in accordance with the analysis of cross-area stLFPs.

**Figure 3:**
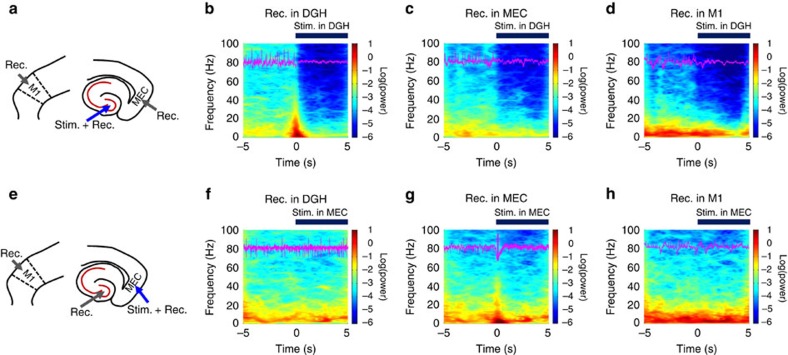
Selectively activating GABAergic interneurons suppress ictal seizure activities. (**a**,**e**) The targeting sites for optical stimulation and electrical recording in kainite mice. (**b**–**d**,**f**–**h**) Mean spectrograms of LFPs recorded in the DGH (**b**,**f**), MEC (**c**,**g**) and M1 (**d**,**h**), respectively (*n*=10 mice). Data were normalized to the maximal log_10_(power) value across the whole 4–100 Hz frequency interval. Purple traces are raw LFP data from representative channels recorded in the DGH (**b**,**f**), MEC (**c**,**g**) and M1 (**d**,**h**), respectively. Light pulses (473 nm, 5 ms pulse duration at 130 Hz) were delivered into the DGH (**a**–**e**) and MEC (**f**–**j**) at time 0. The thick blue line denotes the initial 5 s of stimulation periods.

Previous studies suggested that ictal seizures propagate through a re-entrant loop^3^,^6^,^7^,^8^,^9^, so we hypothesized that decreasing neuronal activity in either the DGH or MEC might interrupt the propagation of ictal discharges. We calculated the LFP power in multiple brain regions of kainate-treated mice simultaneously, to test the inhibitory effect of DGH and MEC GABAergic INs on seizures. Neuronal activity in the primary motor cortex (M1) was also recorded as an indicator of behavioural seizures^29^. After the onset of ictal seizures, the LFP power in the DGH, MEC and M1 all significantly increased within a broad frequency range from 4 to 100 Hz (*n*=10 mice; Fig. 3). Light delivery to the DGH caused a significant decrease in local LFP activity across the whole frequency interval, as well as in the MEC and M1 (*n*=10 mice; Fig. 3a–d; Supplementary Fig. 6), with activity levels reduced to non-kainate baselines. Although activating MEC GABAergic INs suppressed local LFP activity, it showed no significant effect on the activity of DGH or M1 neurons (*n*=10 mice; Fig. 3e–h; Supplementary Fig. 7).

To further localize the affected brain regions under optogenetic stimulation, we injected AAV-DIO-ChR2 virus into the DGH of GAD-Cre mice. A similar result to that in VGAT-ChR2 mice was observed, confirming the contribution of DGH GABAergic INs to seizure inhibition (*n*=5 mice; Supplementary Fig. 8). We also tested whether bidirectional optogenetic modulation (excitation or inhibition) of DGH INs could suppress seizures. However, no significant influence on ictal discharges was observed during optogenetic inhibition of GAD-NpHR-expressing neurons in DGH (*n*=3 mice; Supplementary Fig. 9). This can be explained that the DGH INs mainly show a direct inhibitory effect on local PNs (Supplementary Fig. 3); therefore, optogenetic inhibition of INs could not stop seizures^19^. These results provide direct evidence that the propagation of ictal discharges could be instantly inhibited by selectively activating only DGH, not MEC, GABAergic INs. This also implies that DGH PNs may dominate ictal propagation in the HP–EC circuitry.

### Activating DGH GABAergic INs stops ictal propagation

**Figure 4:**
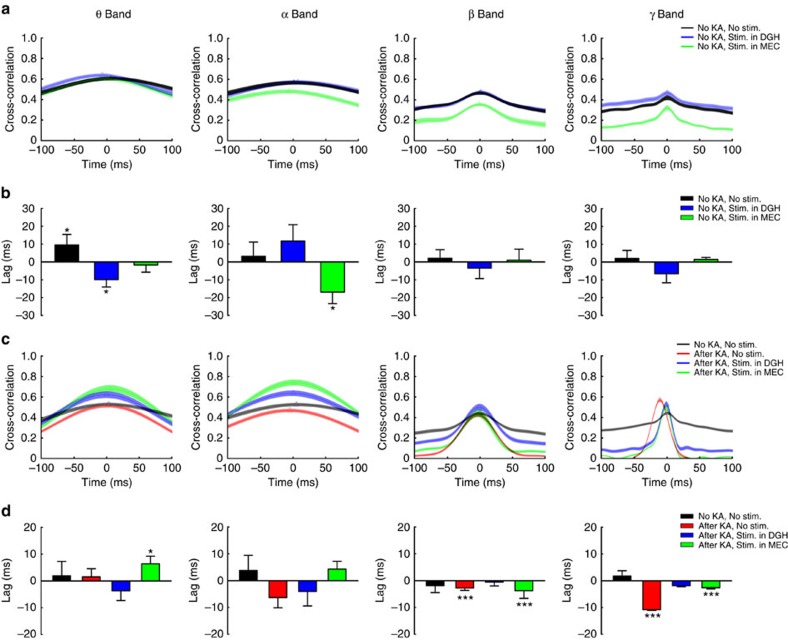
The cross-correlation of instantaneous amplitudes of field potential oscillations between DGH and MEC indicates the propagating direction of ictal seizures. (**a**,**b**) The average distributions (**a**) and lags (**b**) of MEC–DGH cross-correlation peaks during normal state (black traces, *n*=3 mice), stimulation in the DGH (blue traces, *n*=3 mice) and stimulation in the MEC (green traces, *n*=3 mice), respectively. (**c**,**d**): The average distributions (**c**) and lags (d) of MEC–DGH cross-correlation peaks of kainate mice during normal state (black traces, *n*=5 mice), ictal state (red traces, *n*=5 mice) and stimulation in the DGH (blue traces, *n*=5 mice) and MEC (green traces, *n*=5 mice). Triangles represent the maximum points. Error bars represent s.e.m. Stars represent significant differences (*P<0.05, **P<0.01, ***P<0.001, one-sample *t*-test).

To further identify the propagation direction of ictal discharges and investigate the influence of optogenetic modulation on the spread of seizures in the HP–EC structures, we analysed the cross-correlation of instantaneous amplitudes of field potential oscillations^30^. The instantaneous amplitudes of the LFPs recorded in DGH and MEC were cross-correlated, and the average lags and distributions of lags in the cross-correlation peaks were calculated (Fig. 4). In normal conditions, only weak peaks were observed in the theta-, alpha-, beta- and gamma-band cross-correlations (Fig. 4a). The distributions of MEC–DGH lags in all frequency bands were not significantly different from zero (*P*>0.05, one-sample *t*-test, *n*=3 mice; Fig. 4b), suggesting no leading relationship between these two brain regions. In the ictal period, however, strong, sharp cross-correlation peaks emerged, especially in the beta and gamma bands (Fig. 4c), and the distributions of lags were significantly different from zero (*P*<0.001, one-sample *t*-test, *n*=5 mice; Fig. 4d). This result directly suggests a feed-forward propagation of ictal discharges from the DGH to MEC, in accordance with the analysis of cross-area stLFPs^30^,^31^.

We then tested whether selectively exciting GABAergic INs could change the beta–gamma-band activity flow of ictal seizures in HP–EC structures. We noticed that optical stimulation partially changed the distribution of MEC–DGH lags in the theta–alpha band, but did not influence beta–gamma-band information flow during normal state (Fig. 4a,b). During ictal seizures, the distributions of lags across the whole frequency intervals were not significantly different from zero when activating DGH GABAergic INs (*P*>0.05, one-sample *t*-test; Fig. 4d), implying an inhibition of ictal propagation from the DGH to MEC (Supplementary Figs 10 and 11). However, when activating MEC GABAergic INs, the distributions of lags in the beta and gamma bands remained significantly different from zero (*P*<0.001, one-sample *t*-test; Fig. 4d), suggesting that ictal discharges still propagate from the DGH to MEC (Supplementary Fig. 11f). Altogether, these findings imply that the DGH may act upstream of the MEC during ictal propagation, and activating DGH GABAergic INs can directly stop the outward spread of seizures in the HP–EC structures.

We have also tested our findings using an amygdala-kainate model^32^,^33^. After 500 nl of KA solution (0.30 mg ml^−1^) was injected into the basolateral complex of the amygdala (BLA), strong, sharp cross-correlation peaks emerged that were significantly negatively shifted from zero, especially in the gamma band (*P*<0.001, one-sample *t*-test, *n*=8; Supplementary Fig. 12). Optogenetic activation of DGH GABAergic INs caused a significant decrease in LFP activity across the whole frequency interval in the DGH and MEC (*n*=5; Supplementary Fig. 13), whereas activating MEC GABAergic INs failed to inhibit DGH seizures, especially in the beta–gamma band (*n*=5; Supplementary Fig. 14). This result implies that DGH neurons are highly susceptible to seizures, while also being dominant over the MEC in ictal propagation in the HP–EC circuitry of BLA kainate mice.

### Activating DGH INs decreases cross-area coherence

**Figure 5:**
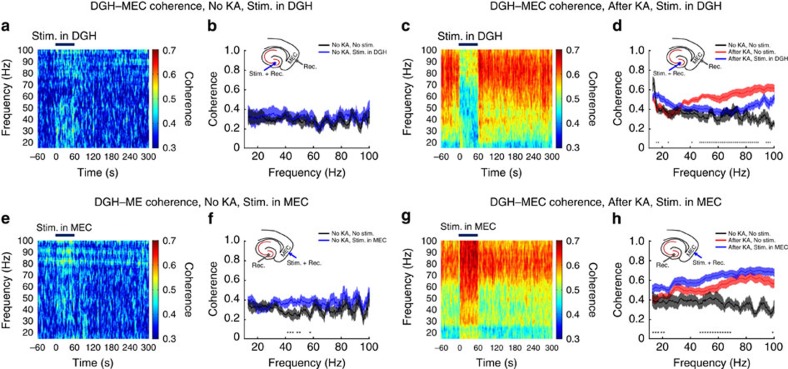
Selectively activating DGH GABAergic interneurons decreases beta–gamma-band LFP coherence. (**a**,**b**,**e**,**f**) LFP coherence spectrum (**a**,**e**) and mean coherence (**b**,**f**) between the DGH and MEC before kainate administration (*n*=10 mice). (**c**,**d**,**g**,**h**) LFP coherence spectrum (**c**,**g**) and mean coherence (**d**,**h**) between the DGH and MEC after kainate administration (*n*=10 mice). Light pulses (473 nm, 5 ms pulse duration at 130 Hz) were delivered into the DGH (**a**–**d**) and MEC (**e**–**h**) at time 0.Insets show the targeting sites for optical stimulation and electrical recording. Thick blue lines denote the 60-s stimulation periods. Error bars represent s.e.m. Stars represent significant differences (*P*<0.01, paired *t*-test).

To quantify the influence of optogenetic modulation on synchronization between distant brain regions during ictal seizures, we analysed LFP coherences in the beta and gamma bands. In the normal state, the averages of DGH–MEC coherence were below 0.4 in the beta and gamma bands (Fig. 5). Activating DGH GABAergic INs showed no significant effect on the values of DGH–MEC (Fig. 5a,b) coherence across most of the beta–gamma frequency intervals, while light delivery to the MEC slightly increased the averages of DGH–MEC coherence (Fig. 5e,f). This may be attributed to variation in the firing pattern of MEC PNs from random to regular during optical stimulation (Supplementary Fig. 4f,h). During ictal seizures, the values of DGH–MEC coherence in the gamma band were significantly increased (Fig. 5c,d,g,h), implying that ictal discharges recruit the downstream neuronal networks into a hypersynchronous state^25^.

We then asked whether this aberrant synchronization between the afferent brain regions could be rescued by activating GABAergic INs. Optogenetic stimulation of the DGH caused a sharp decrease in DGH–MEC (Fig. 5c,d) coherence, especially in the gamma band. Interestingly, activating MEC GABAergic INs significantly increased DGH–MEC (Fig. 5g,h) coherence, especially in the low-gamma band, similar to responses before KA treatment (Fig. 5e,f). Besides, we also found that optogenetic stimulation of the DGH sharply decreased DGH–M1 coherence especially in the gamma band, whereas activating MEC GABAergic INs slightly increased MEC–M1 coherence (Supplementary Fig. 15). These results suggest that selectively exciting GABAergic INs in the DGH, but not the MEC, suppressed ictal propagation and downregulated cross-area synchronization. Accordingly, we can conclude that ictal seizures in the MEC and M1 are propagated from the DGH. Activating DGH GABAergic INs re-establishe the E/I balance of local neuronal networks and thus inhibit outward propagation of ictal discharges.

### Cyclic stimulation has a prolonged inhibitory effect

**Figure 6:**
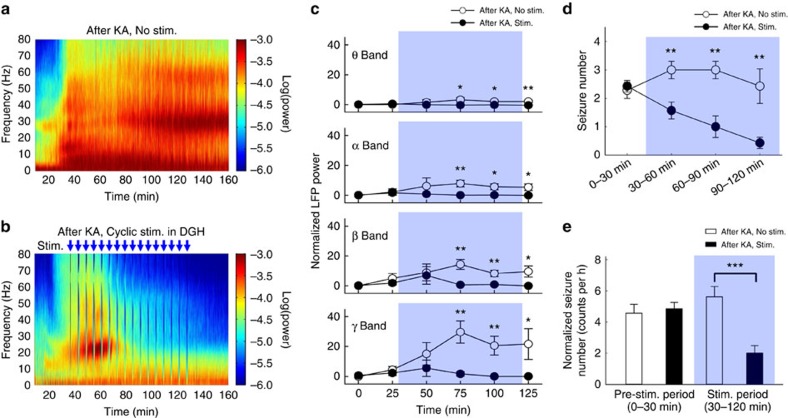
Cyclic optical activation of DGH GABAergic interneurons has a long-lasting inhibitory effect on seizures. (**a**,**b**) Representative examples of spectrograms of LFPs in DGH of non-stimulated (**a**) and optically stimulated (**b**) mice after KA injection. Data were normalized to the maximal log_10_(power) value across the whole 0–80 Hz frequency interval. Each blue arrow represents an optical stimulation cycle (473 nm, 5 ms pulse duration at 130 Hz, 1 min on, 5 min off); a total of 16 cycles were delivered within a 90-min period. Power values were normalized to the total power in the pre-KA period. (**c**) Power quantification of LFPs in the DGH of non-stimulated (*n*=7 mice) and optically stimulated (*n*=8 mice) group after kainate. (**d**) The seizure numbers for Racine grade IV–V behaviours calculated in each 30-min segment during a 120-min period after KA injection. (**e**) Normalized seizure numbers in the stimulated (*n*=7 mice) and non-stimulated (*n*=7 mice) groups. The shaded area shows the 90 min cyclic stimulation period. Error bars represent s.e.m. Stars represent significant differences (*P<0.05, **P<0.01, ***P<0.001, paired *t*-test).

As the distribution of lags shifted towards zero after several light-delivering cycles (Supplementary Fig. 10d), we tested whether cyclic optogenetic activation of DGH GABAergic INs had a long-lasting inhibitory effect on ictal propagation. Approximately 30 min after kainate injection, strong epileptic seizures, lasting for >120 min, were detected in the DGH (Fig. 6a). The theta–gamma-band LFP powers were significantly increased and reached a maximum value at 75 min, followed by a slight decrease (*n*=8 mice; Fig. 6c). After the onset of ictal seizures, cyclic blue light pulses (130 Hz, 5 ms duration, 1 min per cycle) were delivered to activate DGH GABAergic INs. Interstimulation interval was optimized at 5 min due to its long-term inhibitory efficiency (Supplementary Fig. 16). We observed that optical stimulation of the DGH only inhibited ictal seizures after several cycles (Fig. 6b). Detailed analysis showed that LFP activity in the theta and alpha bands did not increase during optical stimulation. The beta and gamma-band LFP powers peaked at 50 min, and then decreased immediately after light delivery.

We also analysed the behaviour of epileptic animals to evaluate the therapeutic effects of optical stimulation. Consistent with electrophysiological recordings, animals showed seizure-related behaviours corresponding to Racine's score III–V shortly after KA treatment^34^ (Supplementary Movie 1). The seizure numbers for Racine grade IV–V behaviours were calculated for each 30-min segment during a 120-min period after KA injection (Fig. 6d). In the non-stimulated group, the average seizure number was 2.29±0.29 (*n*=7, mean±s.e.m.) in period I, increased to 3.00±0.31 in periods II and III (30–90 min), and decreased to 2.43±0.61 in period IV (90–120 min). The average seizure number of the stimulated group (2.43±0.20; *n*=7) in period I was not significantly different from the non-stimulated group (*P*>0.05, paired *t*-test; Fig. 6d,e). However, the animals' epileptic behaviours were quickly suppressed after activation of DGH GABAergic INs (Supplementary Movie 2). The average seizure numbers significantly decreased to 1.57±0.30, 1.00±0.38 and 0.43±0.20 in periods II–IV, respectively (*P*<0.01, paired *t*-test; Fig. 6e). During the entire stimulation period (30–120 min), seizures occurred only 2.00±0.48 times per hour in the stimulated group, significantly less than that in the non-stimulated group (5.62±0.66 times per hour, *P*<0.001, paired *t*-test). Furthermore, 4 of the 11 epileptic mice (36.4%) in the non-stimulated group died of severe seizures during the 120-min test. All animals in the stimulated group survived. This indicates that activating the DGH inhibitory network is sufficient to rescue ongoing behavioural seizures.

**Figure 7:**
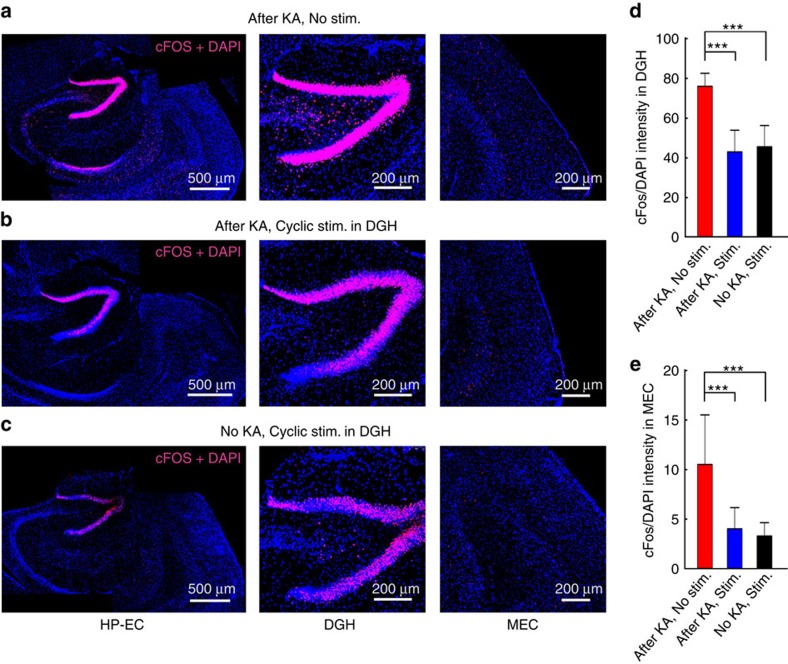
Cyclic optical activation of DGH GABAergic interneurons decreased the expression of cFos in the hippocampal–entorhinal cortex (HP–EC) region. (**a**) cFos expressions in non-stimulated mice 2 h after KA injection (‘After KA, No stim.' group; *n*=20 slices from 4 mice). (**b**) cFos expression in the stimulated mice 2 h after KA injection (‘After KA, Stim.' group; *n*=20 slices from 4 mice). (**c**) cFos expression in the stimulated group without KA injection (‘No KA, Stim.' group; *n*=20 slices from 4 mice). Light pulses (473 nm, 5 ms pulse duration at 130 Hz, 1 min on, 5 min off) were delivered to the DGH during a 90-min period. Colabeling of cFos (red) and DAPI (blue) in the HP–EC regions (left), DGH (middle) and MEC (right) is shown. (**d**,**e**) Averaged cFos/DAPI densities in the DGH (**d**) and MEC (**e**). Error bars represent s.e.m. Stars represent significant differences (*P<0.05, **P<0.01, ***P<0.001, paired *t*-test).

To quantify effects of optogenetic stimulation on the excitability of neurons in HP–EC structures, we also investigated cFos expression in the neurons of epileptic mice (Fig. 7)^35^, which was quantified by cFos/4,6-diamidino-2-phenylindole (DAPI)-staining intensity. Virtually no cells were labelled by cFos in the hippocampal regions of animals before KA injection (Supplementary Fig. 17). Two hours after KA treatment, strong cFos labelling was observed throughout the hippocampal formation, particularly in the DGH regions (‘After KA, No stim.' group; *n*=20 slices from 4 mice, Fig. 7a). This also implies that DGH neurons are highly sensitive to kainate. However, after cyclic optogenetic stimulation, cFos expression significantly decreased in the DGH (‘After KA, Stim.' group; *n*=20 slices from 4 mice, *P*<0.001, paired *t*-test; Fig. 7b,d). Optogenetic stimulation alone could also induce cFos expression in the DGH (‘No KA, No stim.' group; *n*=20 slices from 4 mice; Fig. 7c), perhaps attributable to excitation of DGH GABAergic INs. Expression of cFos was also significantly decreased after optogenetic stimulation in the MEC of epileptic mice (*P*<0.001, paired *t*-test; Fig. 7e). Detailed analysis revealed that cFos density in both the DGH and MEC showed no significant difference between the stimulated groups (‘After KA, Stim.' versus ‘No KA, Stim.', Fig. 7d,e). Despite possible side effects during optogenetic stimulation^16^, cFos-staining results suggest that selectively activating DGH GABAergic INs can notably decrease excitability of neurons in the hippocampal structures. These findings correspond with electrophysiological and behavioural analyses, confirming that DGH dominates the propagation of ictal discharges in a feed-forward HP–EC circuit.

## Discussion

In this study, we used precisely timed, cell-type-specific *in vivo* optogenetic methodologies to dissect the direction of ictal propagation in HP–EC structures. To the best of our knowledge, this is the first description of multi-functional neural probes for drug delivery, optical stimulation and electrical recordings in multiple brain regions in KA-treated animals. By investigating the cross-area multi-unit stLFPs and the cross-correlation of instantaneous amplitudes of LFPs, we found a feed-forward propagation pathway of ictal discharges from the DGH to MEC, instead of a re-entrant loop^3^,^6^,^7^,^8^,^9^,^10^. We demonstrated that selectively activating DGH GABAergic INs significantly inhibited ictal discharges in the DGH, MEC and M1, while optical stimulation in the MEC suppressed seizures only in local networks, not in the DGH or M1. These data provide strong, direct evidence that the DGH dominates the propagation of ictal discharges. Furthermore, we also found that cyclic light delivery to the DGH shows a long-lasting inhibitory effect on ictal seizures, significantly reducing the frequency of behavioural seizures in freely moving animals. Our finding implies that DGH GABAergic INs can directly influence the propagation of seizures in the HP–EC circuitry, which may be a potential therapeutic target for TLE.

Although network dynamics in the hippocampal formations during seizures have been previously investigated^3^,^6^,^7^,^8^,^9^,^15^,^36^, a gap between current knowledge and the understanding of the exact ictal propagation direction in the HP–EC structures still remains. Bridging this gap requires understanding the precise causal relationship between the activities of particular neuron populations and their contribution to ictal discharges in real time at the organismal level^1^. In this study, we induced acute status epilepticus by direct microinjection of KA into the right dorsal hippocampus of VAGT-ChR2 mice^20^. The activity of multi-unit bursts and LFPs in the DGH and MEC were significantly increased after the onset of ictal seizures, suggesting hyperexcitability of the affected neurons. We then performed highly temporally precise analyses of the correlations between the DGH and MEC. Our results show a hypersynchronization between the DGH and MEC during ictal seizures, evidenced by the aberrant strong peaks in the cross-area stLFPs. We found that average peak latencies in DGH–DGH, DGH–MEC and MEC–MEC stLFPs were positive, while that in the MEC–DGH was negative. This result directly suggests a feed-forward propagating direction of DGH spikes→DGH LFPs→MEC spikes→MEC LFPs (Fig. 1o). It also implies that activity of hypersynchronous DGH neurons generated ictal discharges in the DGH and MEC. This hypothesis was further confirmed by findings that light delivery to DGH INs instantly suppressed seizures locally, in the MEC, and in the M1 and significantly decreased DGH–MEC and DGH–M1 coherences, while activating MEC GABAergic INs showed no significant inhibitory effect on ictal activity. In addition, the cross-correlation of instantaneous amplitudes of field potential oscillations was calculated to identify the major propagating direction between the DGH and MEC. The result showed a dominant feed-forward propagation direction of ictal discharges from the DGH to MEC, which could be instantly suppressed by optogenetic activation of DGH GABAergic INs (Fig. 4d; Supplementary Figs 10 and 11). Interestingly, similar results were also obtained in BLA kainate mice. This suggests that DGH neurons are highly susceptible to seizures and play a crucial role in the propagation of ictal discharges.

In addition, we also quantified cFos expression and behavioural seizures in KA-treated mice both with and without optogenetic stimulation. We found that cyclic optical stimulation in the DGH suppressed ictal seizures after several cycles and resulted in a significant decrease in seizure-induced cFos expression in the hippocampal formation, especially in the DGH (Fig. 7). Optical stimulation also rescued behavioural deficits and significantly reduced the number of seizures in the stimulated group versus the non-stimulated group. It should be noted that 36.4% of the non-stimulated mice died of severe epileptic seizures 30–120 min after KA injection, but no mice in the stimulated group died. As recent studies have suggested that epilepsy may increase the risk of sudden unexpected death^37^,^38^, this result suggests that optogenetic activation of DGH GABAergic INs may suppress the development of TLE seizures and increase the survival rate of animals suffering from severe epileptic seizures.

In conclusion, our findings support the hypothesis that DGH PNs are critically involved in TLE, and that they generate outward-spreading epileptic seizures that propagate to the MEC and other brain regions, including the M1. Targeting DGH GABAergic INs may thus be a more practicable approach to TLE treatment. The optimized optical stimulus parameters in this study are similar to those used in deep brain stimulation (DBS), which may provide insight into the mechanism for the therapeutic effects of DBS^39^,^40^. Moreover, as hippocampal–entorhinal cortex circuitry is also associated with other neuropsychiatric disorders including emotional and cognitive impairments, the methodology and findings of this work may also contribute to that research.

## Methods

### Fabrication of optogenetic probes

Multi-channel optrode arrays, each containing 1 optical channel and 8 twisted tetrodes (32 channels), were fabricated from optical fibres (200 μm diameter, numerical aperture=0.37) and 12 μm diameter formvar-coated nickel chromium wires using a custom-made optrode mould for *in vivo* studies. Each tetrode was threaded through a silica tube (75 μm inner, 152 μm outer diameter). In the optrode array, eight tetrodes were arranged around an optical fibre; the spacing between each tetrode and the optical fibre was ∼100 μm. To ensure illumination of the recorded neurons, the tips of the recording sites were ∼300 μm deeper than the optical fibre. Recording sites were plated with platinum to reduce impedance to <500 kΩ (at 1 kHz in artificial cerebrospinal fluid) before use. Approximately 2 mm of the insulation was removed from one end of each microelectrode using a brief flame, and each microelectrode was soldered into separate slots of a standard 36-pin electrode connector. Two pairs of silver microwires (100-μm diameter, 99.95% pure) were then soldered into the electrode connector as ground and reference electrodes, respectively. One end of the optical fibre was fixed onto a custom-made optical connector and stabilized on the electrode connector using a layer of epoxy.

Drug delivery optrode (doptrode) arrays were fabricated following similar procedures. A silica tube (150 μm inner, 350 μm outer diameter) was fixed to the electrode connector parallel to the optrode; spacing between the silica tube and the optical fibre was ∼450 μm. To localize drug delivery to a target brain area, the tip of the silica tubing was ∼500 μm shallower than the optical fibre. One end of the silica tubing was fixed onto a custom-made drug delivery connector connected to a 25-μl microsyringe.

### Surgery

All experiments were performed in accordance with protocols approved by the Ethics Committee for Animal Research, Shenzhen Institute of Advanced Technology, Chinese Academy of Sciences. VGAT-ChR2(H134R)-EYFP bacterial artificial chromosome transgenic mice were used, with expression of the mhChR2::YFP fusion protein directed to GABAergic INs by the mouse vesicular GABA transporter^28^. GAD2-IRES-Cre transgenic mice were purchased from Jackson laboratories (repository number: 010802). Mice were housed under controlled conditions (ambient temperature 24±1 °C, humidity 50–60%, lights on from 0800 to 2000 hours) with food and water *ad libitum*. Male VGAT-ChR2 mice, ∼6–8 weeks of age, were used for our studies.

Mice were treated with atropine (0.2 mg kg^−1^) 15 min before experiment to overcome breathing problems. General anaesthesia was achieved using intraperitoneal administration of urethane (1.5 g kg^−1^). After the animal's head was fixed in a standard stereotaxic frame, the cranium was exposed through a small midline scalp incision. Holes were drilled through the skull, and different neural probes were directed towards the brain at the following stereotaxic coordinates: the optrode tip of the doptrode array at anteroposterior (AP)−2.06 mm, mediolateral (ML)−1.35 mm and dorsoventral (DV)−2.10 mm for optical stimulation and recording in the DGH; the silica tubing of the doptrode array at AP −2.06 mm, ML −1.80 mm and DV −1.60 mm for drug delivery in the dorsal hippocampus; optrode array at AP −5.00 mm, ML −3.00 mm and DV −4.00 mm for optical stimulation and recording in the MEC; electrode array at AP +1.80 mm, ML −1.80 mm and DV −1.80 mm at a 20° angle for recording in the primary motor cortex (M1).

To characterize the onset and propagation of hippocampal seizures, an acute seizure model was used, following previous studies^20^. KA was dissolved in PBS at 0.30 mg ml^−1^, and then 500 nl of KA solution was unilaterally injected into the dorsal hippocampus via the doptrode array using a micropump.

### Electrophysiological recording and optical stimulation

After the onset of ictal seizures (∼30 min after KA injection), a 473-nm blue laser controlled by an analogue input was used for VGAT-ChR2 activation. The laser was conducted into the implanted optrode array using an optical fibre (200 μm diameter) terminated with a custom-made optical connector. Blue light was delivered at a power of 10 mW with a cycling stimulation mode (5 ms pulses at 130 Hz, 1 min on, 5 min off) following previous work in DBS^41^. A detailed experimental design, including optogenetic stimulation paradigms, is shown in Supplementary Fig. 2a.

### Data analysis

Wideband signals were recorded at 40 kHz and LFP signals at 1 kHz. Spike activity was extracted from wideband signals using standard spike sorting routines (Offline sorter, Plexon, USA). The wideband signals were high-pass filtered (300 Hz) with a Bessel filter for detection of spikes. The threshold for spike detection was set to −4.5 s.d.'s, and spike waveforms were measured in a time window 1,400 μs long and beginning 150 μs before threshold crossing. Principal component values were calculated for the unsorted aligned waveforms. A group of waveforms was considered to be generated from one single unit if it was distinct from other clusters. The dead time was set to 1 ms, so each cluster had to exhibit a refractory period >1 ms. The L ratio and isolation distance were used to quantify separation between identified neurons. The L ratio measures the degree of noise contamination of a cluster, a smaller value implies a lower degree of noise contamination. The isolation distance estimates the average distance expected between a cluster and an equal ensemble of spikes outside the cluster, and a bigger value implies a well-isolated cluster. Low values for L ratio and high values for isolation distance were indicative of good cluster separation. The threshold of the L ratio and isolation distance was set to 0.2 and 18, respectively. Units with an L ratio >0.2 and an isolation distance <18 were excluded from the subsequent analysis.

Data analyses were performed with custom software written in Matlab and Neuroexplorer. To characterize putative INs and pyramidal neurons, two features of the extracellular waveform, the peak-to-peak time and the half width of the spikes, were calculated. Neurons with narrow peaks were regarded as putative inhibitory INs, whereas neurons with wide peaks as putative excitatory principal cells. Auto-correlogram histograms and peri-event raster plots were created using Neuroexplorer. For the peri-event raster plot, optical stimulation-induced neural activity was calculated by comparing the firing rate 120 s after stimulus onset with the firing rate recorded during the 60 s period before stimulus onset, using a *z*-score transformation. *Z*-score values were calculated by subtracting the average baseline firing rate over the 60 s preceding stimulus onset and dividing by the baseline s.d.

To calculate LFP powers, the raw LFPs were first filtered in different bands (theta, 4–8 Hz; alpha, 8–12 Hz; beta, 12–30 Hz; gamma, 30–80 Hz). The squared values of the filtered signals 60 s before and 300 s after stimulation (10 s bins) were calculated for each band. The powers before stimulation were averaged over the bins (6 bins, 60 s). The power in each bin after stimulation was compared with the averaged power before stimulation by one-side paired *t*-test to investigate whether stimulation reduced LFP power. The time frequency analysis was conducted using the Smoothed Pseudo Wigner-Ville method.

The optical stimulation-induced multi-unit activity was divided into 1 ms bins. For the stLFP calculation, LFPs 200 ms before and 200 ms after the spike onset were measured. The spikes in the original data set were randomly perturbed on an interval of ±200 ms within 10 ms bins to form the jittered data set. The process was repeated independently 1,000 times to form the data sets. The peak of the stLFP was identified as the first point at which absolute value exceeded four times the threshold of the stLFP. The multi-taper method of the Chrounux analysis package was used for the time frequency and coherence analysis. The value was calculated using a 1-s window, 3 time–bandwidth product (NW) and 5 tapers.

To determine the propagation direction of ictal seizures in the HP–EC circuitry, cross-correlation of instantaneous amplitudes of LFPs was analysed following previous studies^27^,^28^. The LFPs were filtered in different bands (theta–gamma bands) and the instantaneous amplitude calculated using the Hilbert transform. The peak of the cross-correlation of the instantaneous amplitudes was then identified, and the time lags between the DGH and MEC were calculated. The significance of the cross-correlation was verified by the bootstrap method, which randomly shifted the instantaneous amplitude 5–10 s 1,000 times to yield a distribution. Propagation was considered to exist if the peak value was >99% of these random peaks. The length of the signal used for calculation was 180 s and the bin size was 10 s.

### Nissl staining

After electrophysiological recordings, animals were killed for Nissl fluorescent staining to verify the localization of implanted neural probes^42^,^43^. The mice were deeply anaesthetized and perfused through the ascending aorta with 0.1 M PBS (pH 7.3) followed by 4% paraformaldehyde in 0.1 M PBS. The brains were quickly removed from the skull and postfixed overnight in the same fixative at 4 °C. After thorough rinsing in phosphate buffer, the brains were cryoprotected in 0.1 M PBS containing 25% sucrose for 48 h. The brains were then embedded in optimum cutting temperature compound, snap-frozen at −20 °C and stored at −80 °C.

Coronal sections 35 μm in thickness were obtained using a cryostat microtome. The brain sections were washed in PBT (0.1 M PBS plus 0.1% Triton X-100) for 10 min, followed by two more washes in 0.1 M PBS for 5 min each. Red fluorescent Nissl stain (NeuroTrace Fluorescent Nissl Stains, N-21482, Invitrogen) was applied, diluted 1:200 in 0.1 M PBS and incubated for 20 min at room temperature. Next, the sections were washed once with PBT for 10 min and twice with 0.1 M PBS for 5 min each. After again washing the sections overnight at 4 °C in 0.1 M PBS, they were mounted on glass slides and stored at 4 °C. The samples were observed using a Leica TCS SP5 laser scanning confocal microscope equipped with argon and HeNe lasers, and a 543 diode beam splitter.

### Optical stimulation and seizure scoring *in vivo*

Custom-made drug and light delivery implants, each containing a silica drug delivery tube (150 μm inner, 350 μm outer diameter) and an optical fibre (200 μm diameter, numerical aperture=0.37), were fabricated following similar procedures to doptrode manufacture. The implant was inserted into the brain of a VGAT-ChR2 transgenic mouse and fixed to the skull with dental cement. Animals were allowed to recover for at least 5 days before further experimentation. At least one session of 30 min of video was collected before experimental manipulation and used as the baseline control for behavioural analysis. To induce acute status epilepticus, KA (0.15 mg per 500 nl) was then slowly (in a 5-min time window) injected into the right dorsal hippocampus of the VAGT-ChR2 mouse via the implant. Animals that had at least one seizure behaviour of Racine grade IV or above within 30 min of KA treatment were used for further studies. Cyclic light pulses (473 nm, 10 mW, 130 Hz, 5 ms pulse duration, 1 min on, 5 min off, 90 min in total) were delivered into the DGH of stimulated group mice 30 min after KA injection. Behavioural seizures were evaluated according to Racine's scale^31^. The number of seizures with a Racine score of IV or above (including rearing, rearing and falling, loss of posture or jumping) was counted for each animal.

### Immunohistochemistry for cFos protein

To quantify the increased neuronal activity after induced seizures, the brain sections were processed for cFos immunohistochemistry staining following previous work^35^. Thirty minutes after KA treatment, blue light pulses (10 mW, 5 ms pulses at 130 Hz, 1 min on, 5 min off, 90 min total duration) were conducted via optical fibre to activate VGAT-expressing neurons in the DGH of transgenic mice. Both groups of animals (‘After KA, No stim.' group, *n*=4; ‘After KA, Stim.' group, *n*=4) were killed and perfused 120 min after KA injection. Another group of VGAT-ChR2 mice (‘No KA, Stim.' group, *n*=4), which received no KA treatment, were killed and perfused after 90 min cyclical stimulation. A detailed experimental design is summarized in Supplementary Fig. 2b.

Sequential horizontal sections, 35 μm in thickness, of the HP–EC region and M1 were obtained using a cryostat microtome (Leica CM1950, Germany). The slices were treated with PBS three times for 5 min each and blocked in 10% normal goat serum (005-000-121, Jackson Immuno Research) for 1 h. The samples were then incubated with primary antibodies (1:200; cFos [9F6]rabbit mAb, #2250, Cell Signal Technology) overnight at room temperature, washed with PBS and incubated with DyLight594 goat anti-rabbit (1:100; AlexaFluor 594-conjugated affinpure goat anti-rabbit IgG(H+L), 111-585-003, Jackson Immuno Research) for 1 h. After washing with PBS three times for 5 min each, the sections were mounted onto gelatin-coated slides and cover slipped with signal enhancer (ProLong Gold Antifade Reagent with DAPI, P36935, Invitrogen).

Mounted slices were evaluated for fluorescence under settings for AlexaFluor 488 (green), 543 (red) and 405 (blue) on a Leica TCS SP5 laser scanning confocal microscope equipped with argon and HeNe lasers and a 405 diode beam splitter. The settings of the confocal microscope were kept constant throughout the imaging, such that fluorescence intensities could be compared between animals and imaging days. For slice imaging, each slice was imaged across the HP–EC or M1 regions. ChR2-GFP expression images were taken and their cFos/DAPI content was analysed. Slice preparation and imaging were performed by a researcher unaware of treatment groups.

## Additional information

**How to cite this article**: Lu, Y. *et al.* Optogenetic dissection of ictal propagation in the hippocampal–entorhinal cortex structures. *Nat. Commun.* 7:10962 doi: 10.1038/ncomms10962 (2016).

## Supplementary Material

Supplementary Information Supplementary Figures 1-17

Supplementary Movie 1 Animal shows spontaneous behavioral seizures after Kainic Acid injection.

Supplementary Movie 2 Optogenetic activation of DGH GABAergic interneurons rescues the behavioral deficits of Kainic Acid treated mouse.
