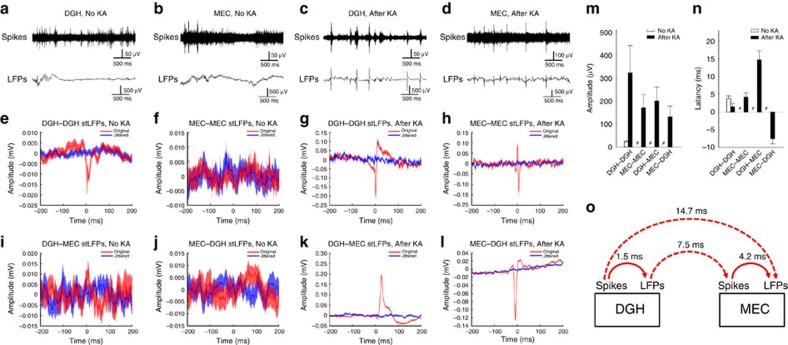# Corrigendum: Optogenetic dissection of ictal propagation in the hippocampal–entorhinal cortex structures

**DOI:** 10.1038/ncomms12019

**Published:** 2016-06-14

**Authors:** Yi Lu, Cheng Zhong, Lulu Wang, Pengfei Wei, Wei He, Kang Huang, Yi Zhang, Yang Zhan, Guoping Feng, Liping Wang

*Nature Communications* 7: Article number:1096210.1038/ncomms10962 (2016); Published 03212016; Updated 06142016

In Fig. 1m of this Article, the *y* axis label has incorrect units and should read ‘Amplitude (μV)'. The correct version of Fig. 1 appears below.

## Figures and Tables

**Figure 1:**