# Supplementary material for: Optogenetic dissection of ictal propagation in the hippocampal–entorhinal cortex structures
Source: Nat Commun. 2016 Mar 21;7:10962. doi: 10.1038/ncomms10962 (PMC4802168; doi:10.1038/ncomms10962)
Supplement: Supplementary Information — Supplementary Figures 1-17 [file ncomms10962-s1.pdf]

Supplementary Figure 1

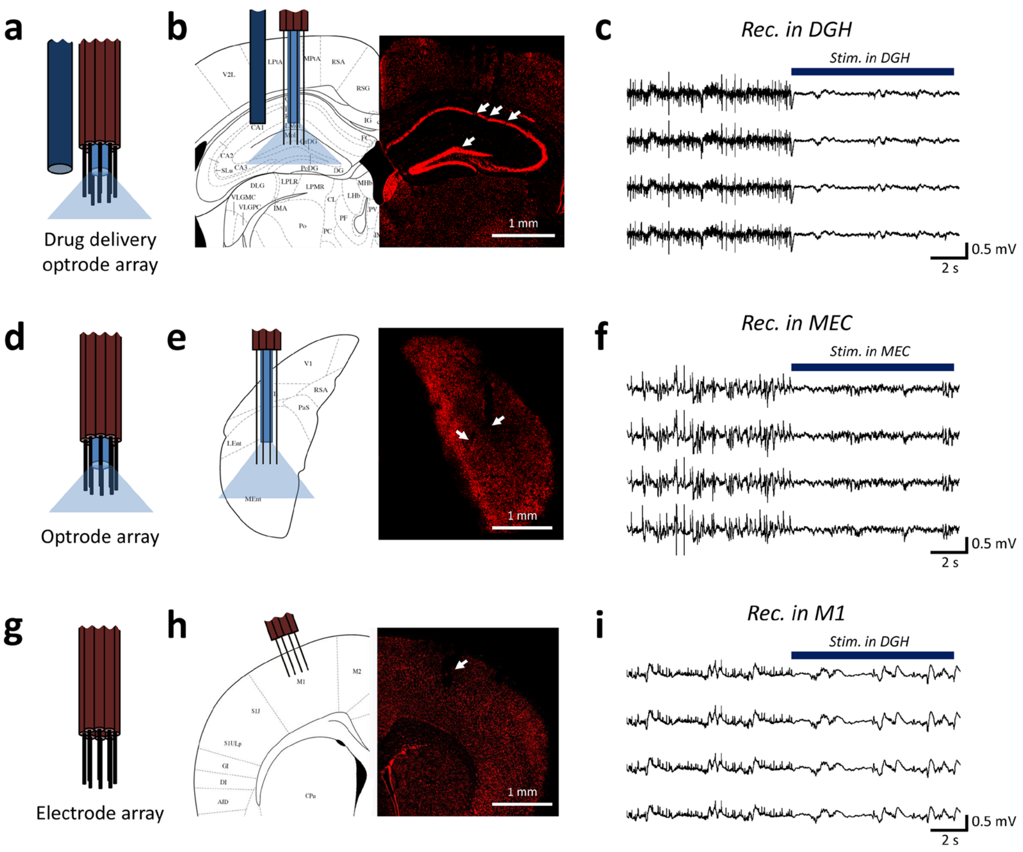

Supplementary Figure 1. Neural probes and implant locations. (**a**): Diagram of 32-channel drug delivery optrode (doptrode) array. Each doptrode array contains a silica tube for drug delivery, an optical fiber for stimulation, and eight tetrodes (32-channel) for electrical recording. (**b**): A Nissl-stained coronal section showing the locations of drug delivery, optical stimulation, and recording sites in hippocampus (white arrows). (**c**): Raw LFPs recorded from the DGH of kainate mouse before and during local light delivery. (**d**): Diagram of 32-channel optrode array. Each optrode array contains an optical fiber for stimulation and eight tetrodes for electrical recording. (**e**): A Nissl-stained coronal section showing the locations of optical stimulation and recording sites in the MEC. (**f**): Raw LFPs recorded from the MEC before and during local optical stimulation. (**g**): Diagram of 32-channel electrode array with eight tetrodes for electrical recording. (**h**):

Nissl-stained coronal section showing locations of recording sites in the M1. (**i**): Raw LFPs recorded from the M1 before and during optical stimulation in the DGH. No significant changes in M1 LFPs were observed when activating the MEC GABAergic interneurons.

Supplementary Figure 2

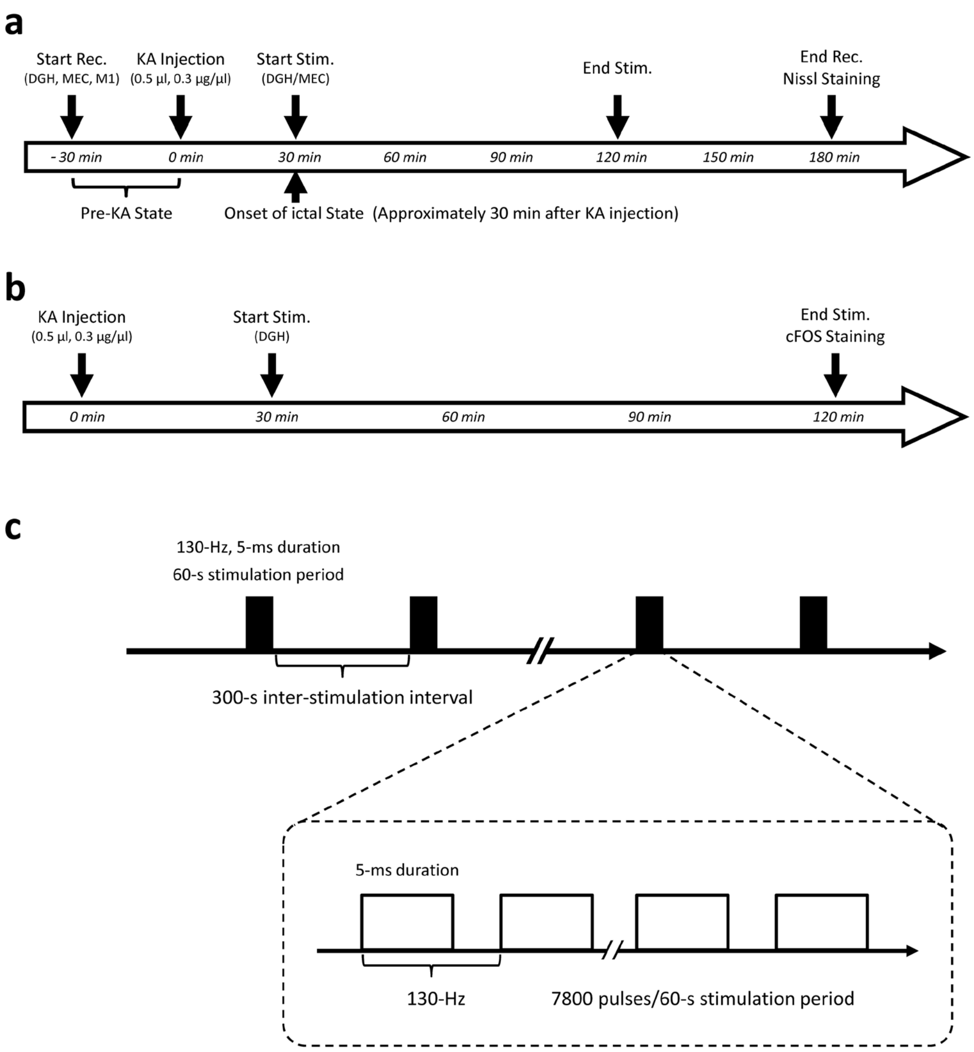

Supplementary Figure 2. Experimental design. (**a**): A timeline showing sequence of events in the recording and optical stimulation experiment. Continuous electrophysiological recordings were carried out in multiple brain areas in VGAT-ChR2(H134R)-EYFP BAC transgenic mice for at least 30 min prior to KA injection. After the onset of ictal seizures, 473 nm light pulses (5 ms pulse duration at 130 Hz) were delivered. Recordings were ended approximately 180 min after kainate injection, and animals were then sacrificed for Nissl staining to verify the localization of implants. (**b**): A timeline of cFos staining experiments. Thirty min after KA injection, cyclic blue light pulses

(5 ms pulse duration at 130 Hz, 1 min on, 5 min off) were conducted by optical fiber into the DGH for 90 min. The animals (control group, n = 10; stimulation group, n = 10) were sacrificed and perfused 120 min after KA injection. (**c**): The optical stimulation waveform. Blue light pulses (130 Hz, 473 nm, 10 mW) with 5 ms pulse duration were delivered in a 6min stimulation cycle (1 min on, 5 min off) for 90 min.

Supplementary Figure 3

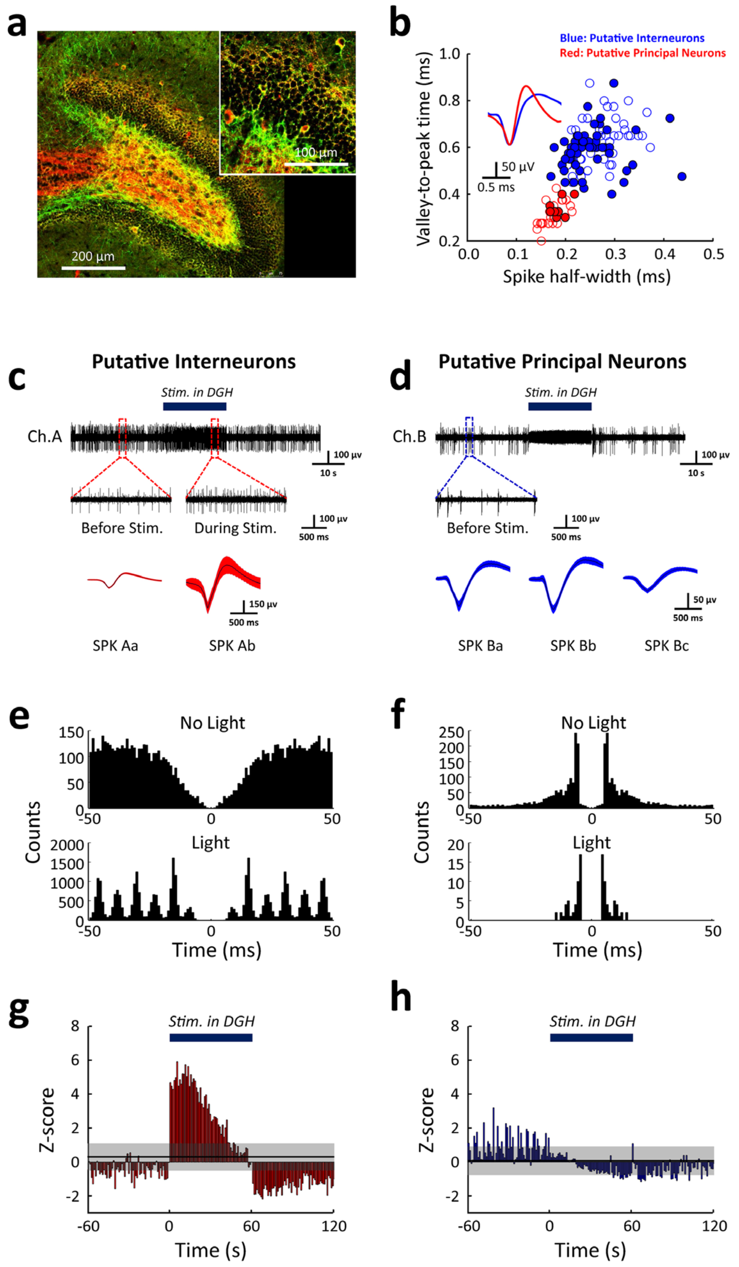

Supplementary Figure 3. Functional characterization of ChR2-EYFP expression interneurons in the DGH of VGAT-ChR2(H134R)-EYFP BAC transgenic mice. (**a**): Co-localization of GAD67 immunoreactivity and endogenous YFP labeling in the DGH. Insets show enlarged views at low fields. The specific expression of ChR2 in GABAergic interneurons was evidenced by the high colocalization of endogenous YFP and GAD67. (**b**): Separation of putative principal neurons (pPNs,

blue, n = 98 neurons) and putative interneurons (pINs, red, n = 35 neurons) recorded in the DGH. The insets show waveforms of pPNs (blue) and pINs (red), respectively. All the neurons activated by 130 Hz blue light were identified to be pINs (red filled circle, n = 8 neurons), whereas all the inhibited neurons were pPNs (blue filled circle, n = 52 neurons). (**c, d**): Representative examples of signals recorded from the DGH in control VGAT-ChR2 mouse via different optrode channels under optical stimulation. The single units recorded were classified into pINs (red traces) and pPNs (blue traces) according to spike half-width and valley-to-peak time width features. (**e, f**): The autocorrelograms (upper panels, no light; lower panels, light) of pINs (e) and pPNs (f) recorded from the DGH. The firing rates of pINs obviously increased and the interspike intervals (~7.7 ms) became more regular during stimulation (e). The firing rates of pPNs significantly decreased during stimulation, but the bursting firings of pPNs did not change (f). (**g, h**): Z-score normalized firing rates of pINs (g) and pPNs (h). Thick blue lines denote 60 s stimulation periods; the shaded area shows the upper and lower limits of the confidence interval. Activating GABAergic interneurons significantly suppressed the activity of local principal neurons, and the inhibitory effect on pPNs slightly increased during the whole stimulation interval despite the decreased firing rates of pINs.

Supplementary Figure 4

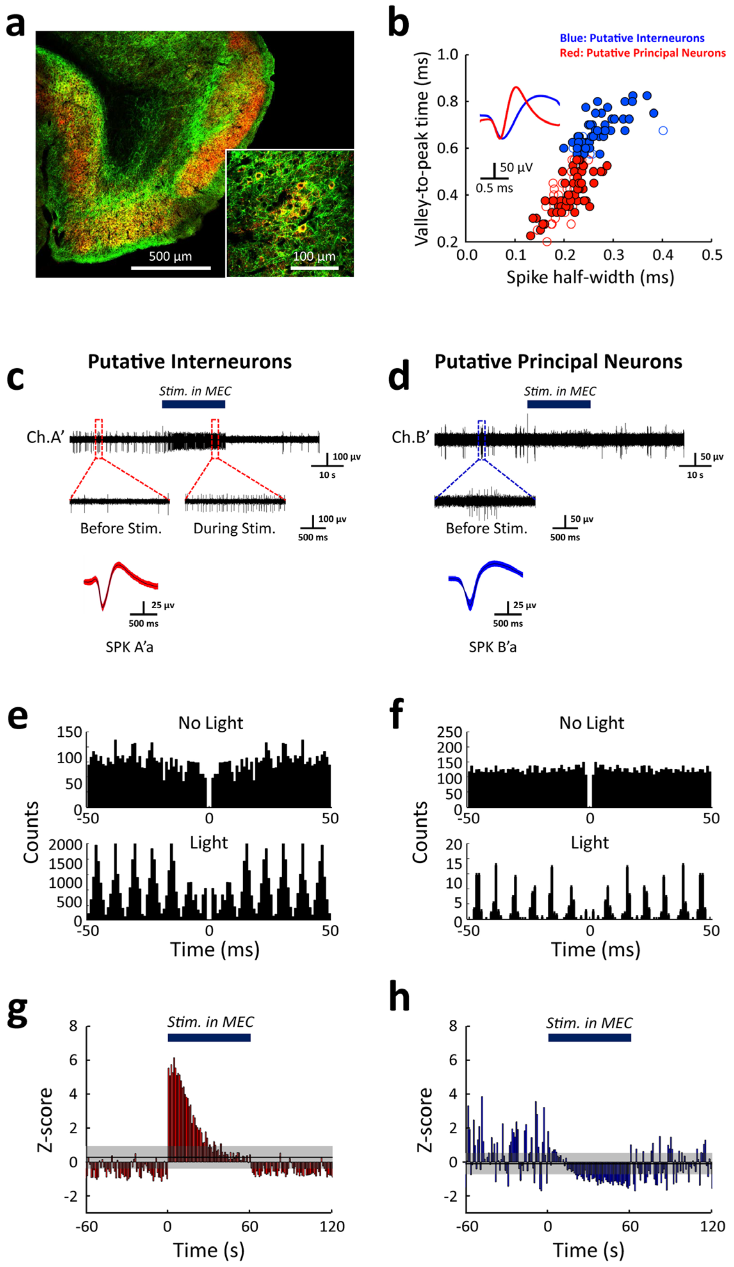

Supplementary Figure 4. Functional characterization of ChR2-EYFP expression interneurons in the MEC of VGAT-ChR2(H134R)-EYFP BAC transgenic mice. (**a**): Colocalization of GAD67 immunoreactivity and endogenous YFP labeling in the MEC. Insets show enlarged views at low fields. The specific expression of ChR2 in GABAergic interneurons was evidenced by the high colocalization of endogenous YFP and GAD67. (**b**): Separation of putative principal neurons (pPNs,

blue, n = 51 neurons) and putative interneurons (pINs, red, n = 93 neurons) recorded in the MEC. The insets show waveforms of pPNs (blue) and pINs (red), respectively. All the neurons activated by 130 Hz blue light were identified to be pINs (red filled circle, n = 45 neurons), while all the inhibited neurons were pPNs (blue filled circle, n = 41 neurons). (**c, d**): Representative examples of signals recorded from the MEC in control VGAT-ChR2 mice in different optrode channels under optical stimulation. The single units recorded were classified into pINs (red traces) and pPNs (blue traces) according to spike half-width and valley-to-peak time width features. (**e, f**): The autocorrelograms (upper panels, no light; lower panels, light) of pINs (e) and pPNs (f) recorded from the DGH. The firing rates of pINs increased, and the interspike intervals (~7.7 ms) became more regular during stimulation (e). The firing rates of pPNs significantly decreased during stimulation, but the spiking pattern of MEC pPNs became more regular, which is highly concordant with the activated local pINs during stimulation and implies increased synchronization in the MEC (f). (**g, h**): Z-score normalized firing rates of pINs (g) and pPNs (h). Thick blue lines denote 60 s stimulation periods; the shaded area shows the upper and lower limits of the confidence interval. Activating GABAergic INs significantly suppressed the activity of local principal neurons, and the inhibitory effect on pPNs slightly increased during the whole stimulation interval despite the decreased firing rates of pINs.

Supplementary Figure 5

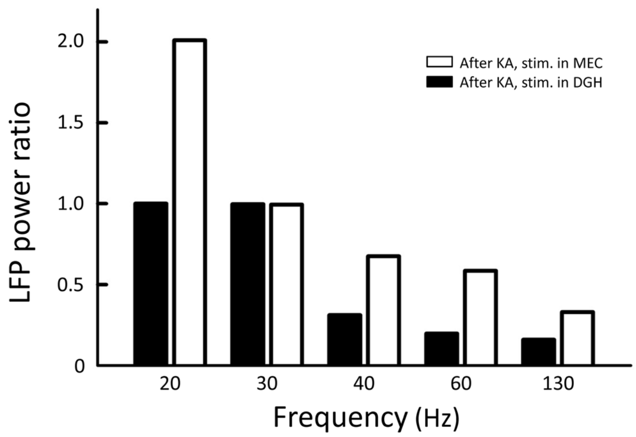

Supplementary Figure 5. The influence of stimulation frequency on seizure inhibition. Light pulses (473 nm, 10 mW, 5 ms pulse duration) were delivered into the DGH and MEC of kainate mice, respectively. Low-frequency optical stimulation (20 and 30 Hz) in the DGH and MEC showed little, or even negative inhibitory effects on seizures, whereas optical stimulation at higher frequencies (> 40 Hz) showed significantly improved inhibitory effects on seizures. Accordingly, 130 Hz was chosen as an optimized stimulation frequency in this study.

Supplementary Figure 6

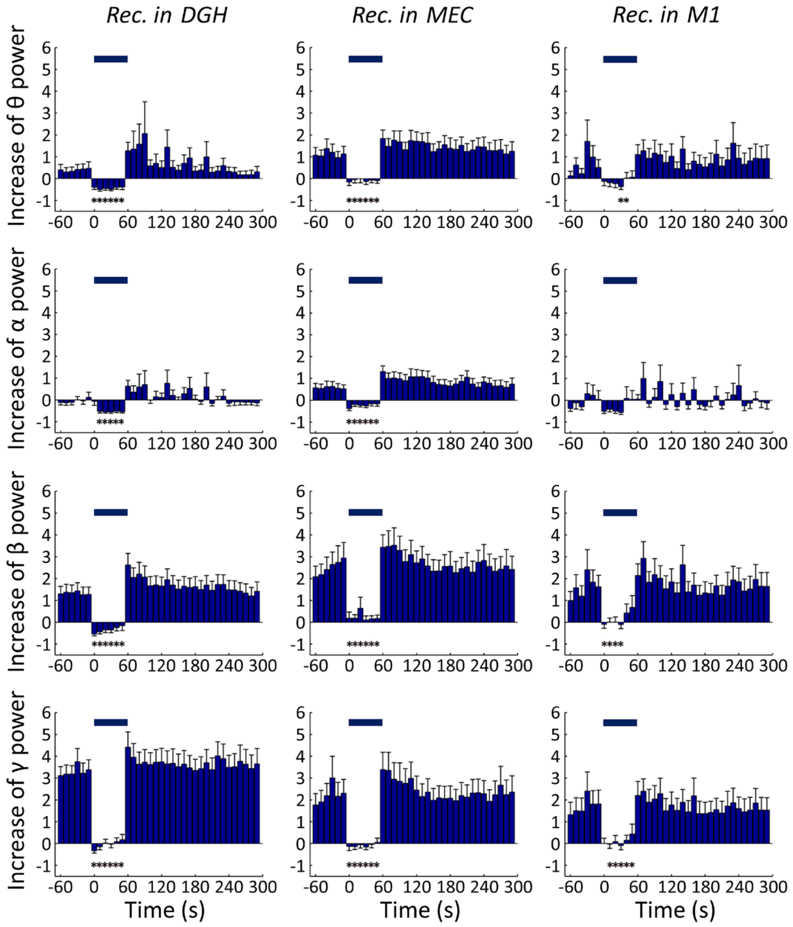

Supplementary Figure 6. Selectively activating DGH GABAergic interneurons suppresses seizure activities in intra-hippocampal kainate mice. Power quantification of DGH (left), MEC (middle), and M1 (right) LFPs before, during, and after optical stimulation (n = 10 mice). Light pulses (473 nm, 5 ms pulse duration, 130 Hz) were delivered to the DGH at time 0. Averaged powers are shown in the theta, alpha, beta, and gamma band (10 s bins). Power values were normalized to the total power in the pre-KA period. Light delivery to the DGH caused a significant decrease in LFP power across the whole frequency interval in local neurons, as well as the MEC and M1. Thick blue lines denote the 60 s stimulation periods. Error bars represent SEM. Stars represent significant differences ($p < 0.01$, paired t-test).

Supplementary Figure 7

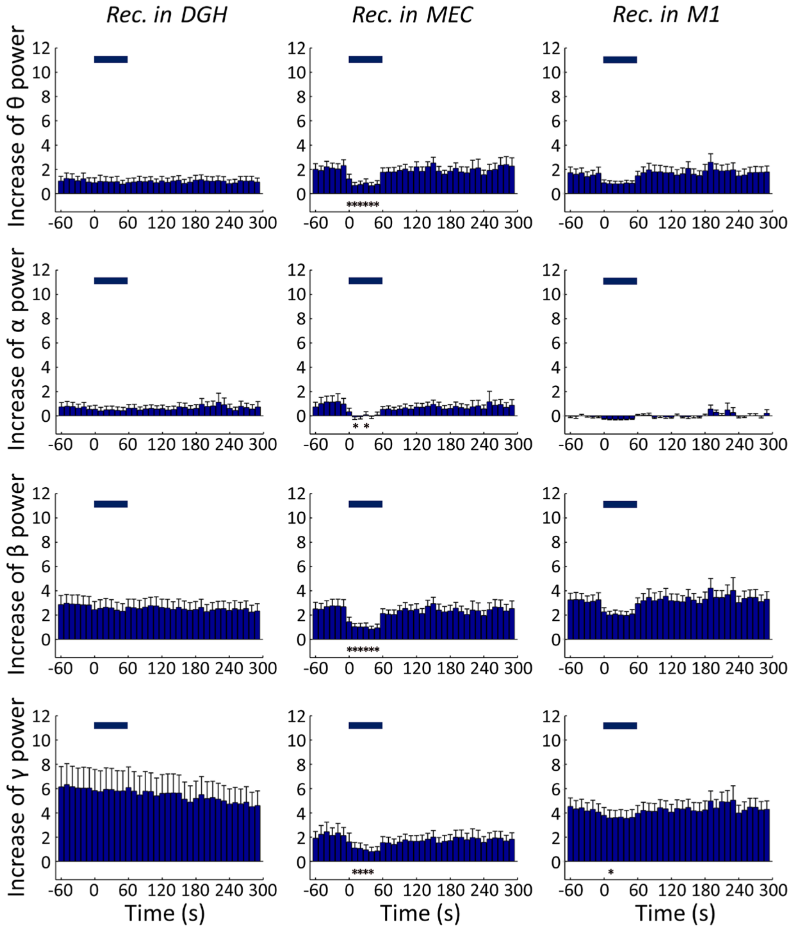

Supplementary Figure 7. Selectively activating MEC GABAergic interneurons could not inhibit ictal seizures in the DGH or M1 in intrahippocampal kainate mice. Power quantification of DGH (left), MEC (middle), and M1 (right) LFPs before, during, and after optical stimulation (n = 10 mice). Light pulses (473 nm, 5 ms pulse duration, 130 Hz) were delivered to the DGH at time 0. Averaged powers were shown in the theta, alpha, beta, and gamma band (10 s bins). Power values were normalized to the total power in the pre-KA period. Activating MEC GABAergic interneurons significantly suppressed local LFP activity, but had no significant effect on seizures in the DGH and M1. Thick blue lines denote 60 s stimulation periods. Error bars represent SEM. Stars represent significant differences ($p < 0.01$, paired t-test).

Supplementary Figure 8

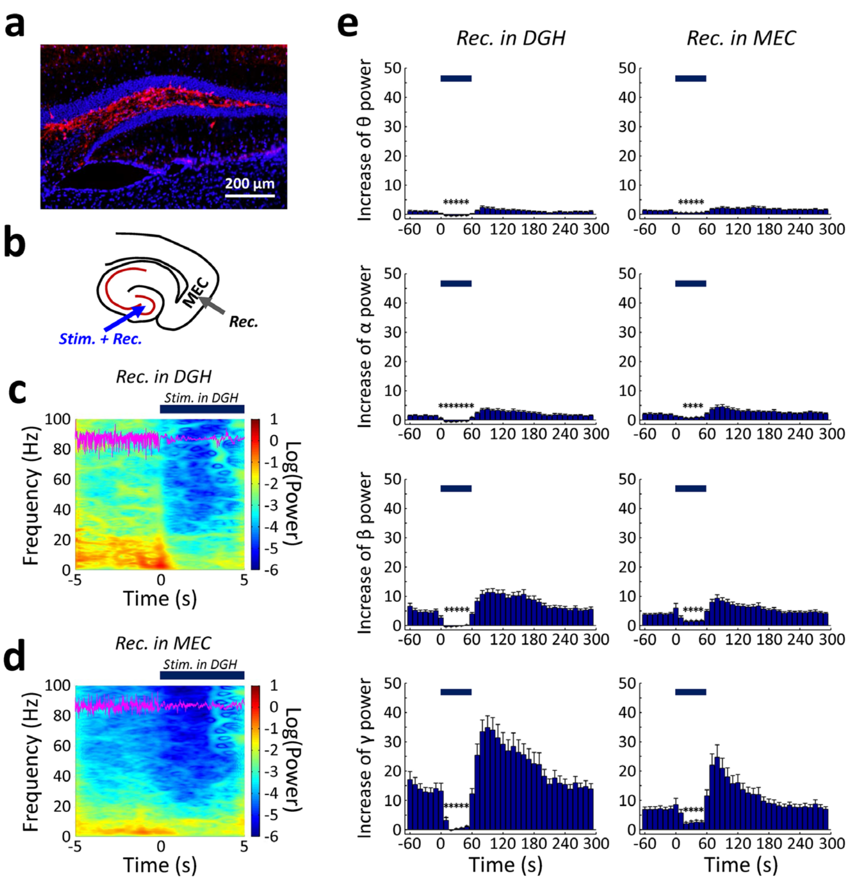

Supplementary Figure 8. Optogenetic activation of GAD-ChR2-expressing neurons in the DGH suppresses seizure activities in intrahippocampal kainate mice. (**a**):Anatomical location of ChR2-mCherry-expressing neurons in the DGH of GAD-Cre mice (red, mCherry; blue, DAPI). AAV-DIO-ChR2 virus was stereotactically injected into the DGH of GAD-Cre mice to localize the brain area stimulated by light pulses. (**b**): Targeting sites for optical stimulation and electrical recording in vivo. (**c-d**): Mean spectrograms of LFPs recorded in the DGH (c) and MEC (d) (n = 5 mice). Data were normalized to the maximal $\log_{10}$(power) value across the whole 4–100 Hz frequency interval. The purple traces were raw LFP data from representative channels recorded in DGH (c) and MEC (d), respectively. Light pulses (473 nm, 5 ms pulse duration, 130 Hz) were

delivered to the DGH at time 0. Thick blue lines denote the initial 5 s of stimulation periods. (**e**): Power quantification of DGH (left) and MEC (right) LFPs before, during, and after optical stimulations (n = 5 mice). Averaged powers were shown in the theta, alpha, beta, and gamma band (10 s bins). Power values were normalized to total power in the pre-KA period. Light pulses were delivered to the DGH at time 0, which caused a significant decrease in LFP power across the whole frequency interval in local interneurons and in the MEC. Thick blue lines denote 60 s stimulation periods. Error bars represent SEM. Stars represent significant differences ($p < 0.01$, paired t-test).

Supplementary Figure 9

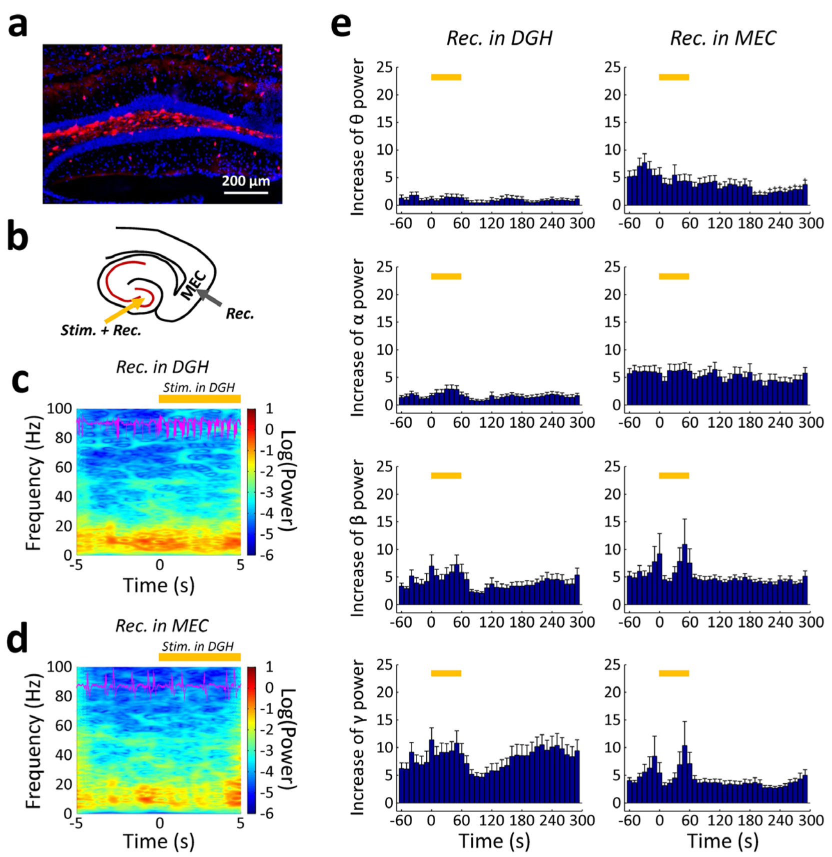

Supplementary Figure 9. Optogenetic inhibition of GAD-NpHR-expressing neurons in the DGH has no significant effect on seizure activities in intrahippocampal kainate mice. (**a**): Anatomical location of NpHR-mCherry-expressing neurons in the DGH of GAD-Cre mice (red, mCherry; blue, DAPI). AAV-DIO-ChR2 virus was stereotactically injected into the DGH of GAD-Cre mice to localize the brain area stimulated by light pulses. (**b**): Targeting sites for optical stimulation and electrical recording in vivo. (**c-d**): Mean spectrograms of LFPs recorded in the DGH (c) and MEC (d) (n = 3 mice). Data were normalized to the maximal $\log_{10}$(power) value across the whole 4-100 Hz frequency interval. Purple traces show raw LFP data from representative channels recorded in the DGH (c) and MEC (d). Light (596 nm) was delivered into the DGH at time 0; thick yellow lines denote the initial 5 s of stimulation periods. (**e**): Power quantification of DGH (left) and MEC

(right) LFPs before, during and after optical stimulation (n = 3 mice). Averaged powers were shown in the theta, alpha, beta, and gamma band (10 s bins). Power values were normalized to the total power in the pre-KA period. Light pulses were delivered to the DGH at time 0. No significant difference in LFP power across the whole frequency interval was observed in local interneurons or in the MEC. Thick yellow lines denote the 60 s stimulation periods. Error bars represent SEM. Stars represent significant differences ($p < 0.01$, paired t-test).

Supplementary Figure 10

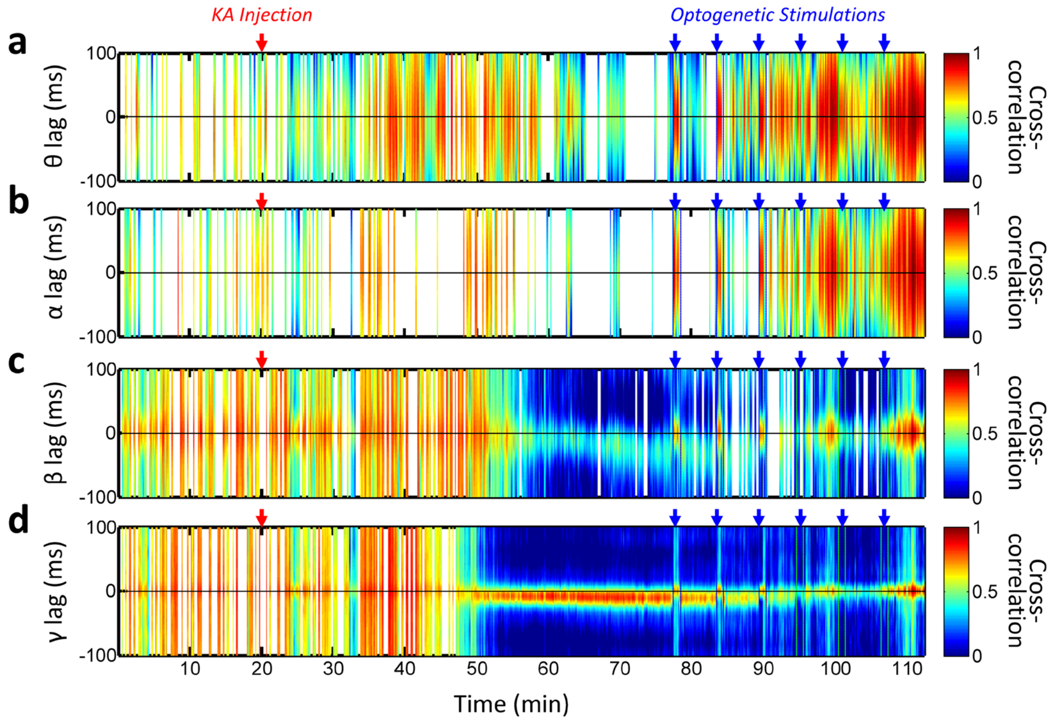

Supplementary Figure 10. A representative amplitude cross-correlation over time between the MEC and DGH in the theta (a), alpha (b), beta (c), and gamma (d) bands. Red arrow represents intrahippocampal KA injection; each blue arrow represents an optical stimulation cycle (473 nm, 5 ms pulse duration, 130 Hz). Warmer colors correspond to higher cross-correlation values. White indicates that the peak value failed to exceed the 99% distribution of the random peaks, suggesting no leading relationship between the MEC and DGH. Note that the lags in the cross-correlation in the gamma band were significant negatively shifted 30 min after KA injection, indicating a directional propagation of ictal seizures from the DGH to MEC. The lags shifted towards zero after several cycles, implying a long-lasting effect of optical stimulation in the DGH.

Supplementary Figure 11

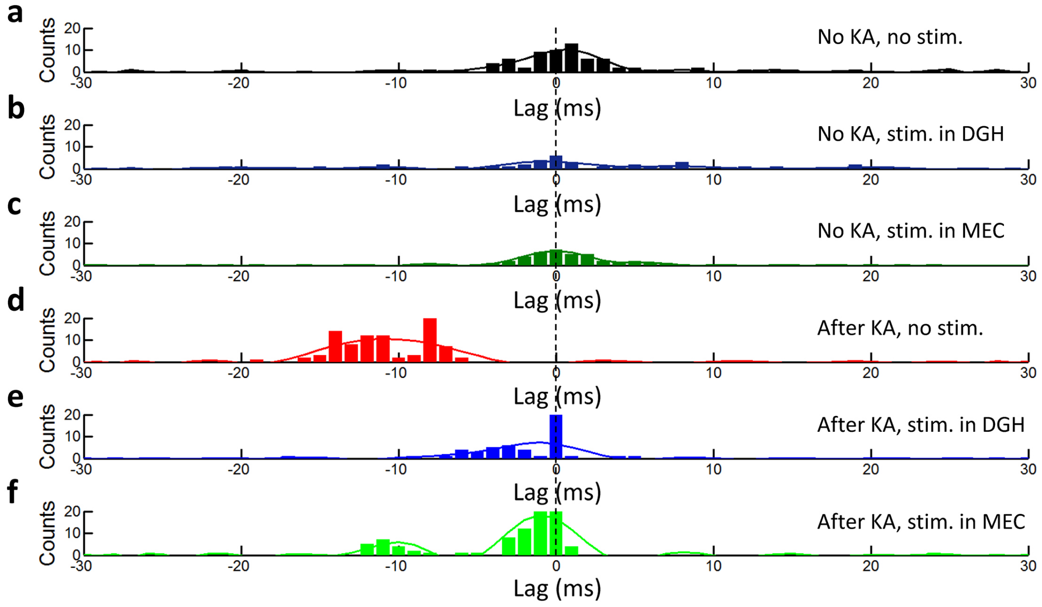

Supplementary Figure 11. Detailed analysis of the distribution of gamma-band MEC-DGH lags. **(a):** Before KA injection, the distribution of gamma band MEC-DGH lags was not significantly different from zero ($p > 0.05$, one-sample t-test), suggesting no leading relationship between the two brain regions. **(b-c):** Optical stimulation in DGH (b) or MEC (c) did not significantly affect the distribution of lags ($p > 0.05$, one-sample t-test). **(d):** After intrahippocampal kainate, the distribution of gamma band lags significantly differed from zero ($p < 0.001$, one-sample t-test) with an average lag value of -11 ms, suggesting a directional propagation of ictal seizures from the DGH to MEC. **(e-f):** Optical stimulation of the DGH shifted the distribution towards zero ($p > 0.05$, one-sample t-test, e), but activation of MEC GABAergic interneurons did not completely eliminate the distribution of gamma band lags at around -11 ms, and the distribution of gamma band lags still significantly differed from zero ($p < 0.001$, one-sample t-test, f), implying the propagation of ictal seizures was not completely hindered.

Supplementary Figure 12

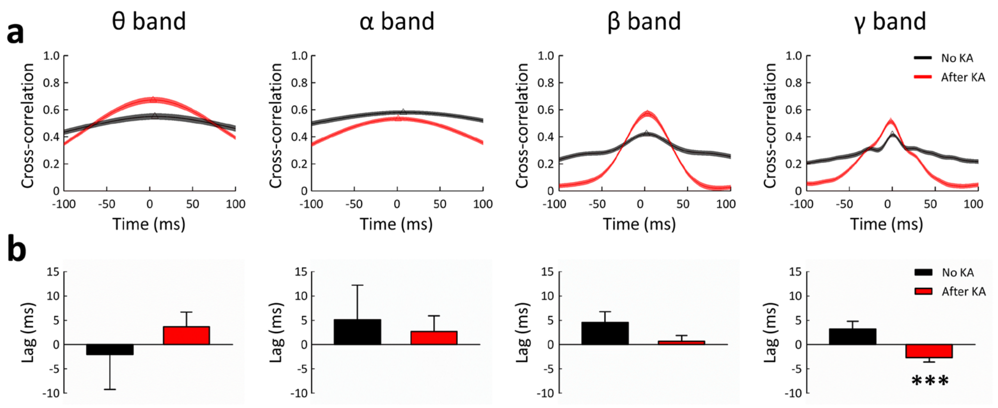

Supplementary Figure 12. The cross-correlation of instantaneous amplitudes of field potential oscillations between the DGH and MEC indicates the propagation direction of ictal seizures. The average distributions (a) and lags (b) of MEC-DGH cross-correlation peaks for normal state (n = 8 mice) and ictal state (n = 8 mice) BLA kainite mice, respectively. Before KA injection, the distributions of theta-gamma band MEC-DGH lags were not significantly different from zero ($p > 0.05$, one-sample t-test), suggesting no leading relationship between the two brain regions. After BLA kainate administration, strong peaks emerged, especially in the beta and gamma bands. The distributions of theta-beta band MEC-DGH lags were not significantly different from zero ($p > 0.05$, one-sample t-test). However, the distribution of gamma band lags significantly differed from zero ($p < 0.001$, one-sample t-test), suggesting a directional propagation of ictal seizures from the DGH to MEC. Triangles represent the maximum points. Error bars represent SEM. Stars represent significant differences (* $p < 0.05$, ** $p < 0.01$, *** $p < 0.001$, one-sample t-test).

Supplementary Figure 13

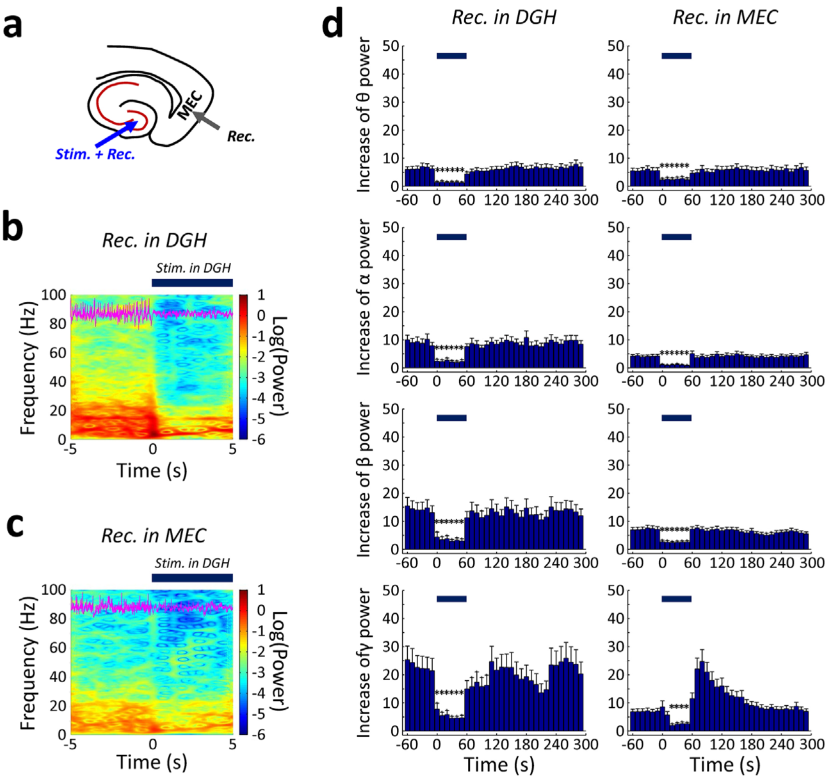

Supplementary Figure 13. Optogenetic activation of DGH GABAergic interneurons inhibits seizure activities in BLA kainate mice. (**a**): The targeting sites for optical stimulation and electrical recording in vivo. KA was stereotactically injected into the BLA of VGAT-ChR2 mice. (**b-c**): Mean spectrograms of LFPs recorded in the DGH (b) and MEC (c), respectively (n = 5 mice). Data were normalized to the maximal $\log_{10}$(power) value across the whole 4–100 Hz frequency interval. Purple traces were raw LFP data from representative channels recorded in the DGH (b) and MEC (c). Light pulses (473 nm, 5 ms pulse duration, 130 Hz) were delivered into the DGH at time 0; thick blue lines denote the initial 5 s of stimulation periods. (**d**): Power quantification of DGH (left) and MEC (right) LFPs before, during, and after optical stimulation (n = 5 mice). Averaged powers were shown in the theta, alpha, beta, and gamma band (10 s bins). Power values were normalized to the total power in the pre-KA period. Light pulses were delivery to DGH at time 0,

which caused a significant decrease in LFP power across whole frequency interval in local interneurons and in the MEC. Thick blue lines denote 60 s stimulation periods. Error bars represent SEM. Stars represent significant differences ($p < 0.01$, paired t-test).

Supplementary Figure 14

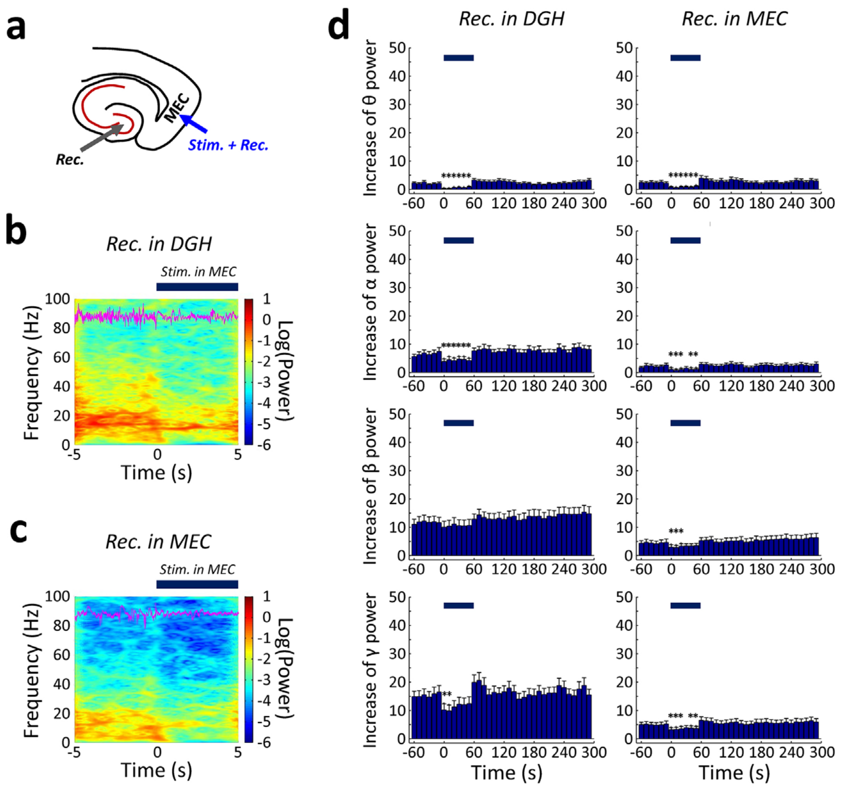

Supplementary Figure 14. Selectively activating MEC GABAergic interneurons could not inhibit ictal seizures in BLA kainate mice. (**a**): The targeting sites for optical stimulation and electrical recording in vivo. KA was stereotactically injected into the BLA of VGAT-ChR2 mice. (**b-c**): Mean spectrograms of LFPs recorded in DGH (b) and MEC (c) (n = 5 mice). Data were normalized to the maximal log_{10}(power) value across the entire 4–100 Hz frequency interval. Purple traces are raw LFP data from representative channels in the DGH (b) and MEC (c). Light pulses (473 nm, 5 ms pulse duration, 130 Hz) were delivered to the DGH at time 0; thick blue lines denote the initial 5 s of stimulation periods. (**d**): Power quantification of DGH (left) and MEC (right) LFPs before, during, and after optical stimulations (n = 5 mice). Averaged powers were shown in the theta, alpha, beta, and gamma band (10 s bins). Power values were normalized to the total power in the pre-KA period. Light pulses were delivered to the DGH at time 0. Optical stimulation did not significantly

decrease the LFP power in the DGH or MEC. Thick blue lines denote the 60 s stimulation periods.

Error bars represent SEM. Stars represent significant differences ($p < 0.01$, paired t-test).

Supplementary Figure 15

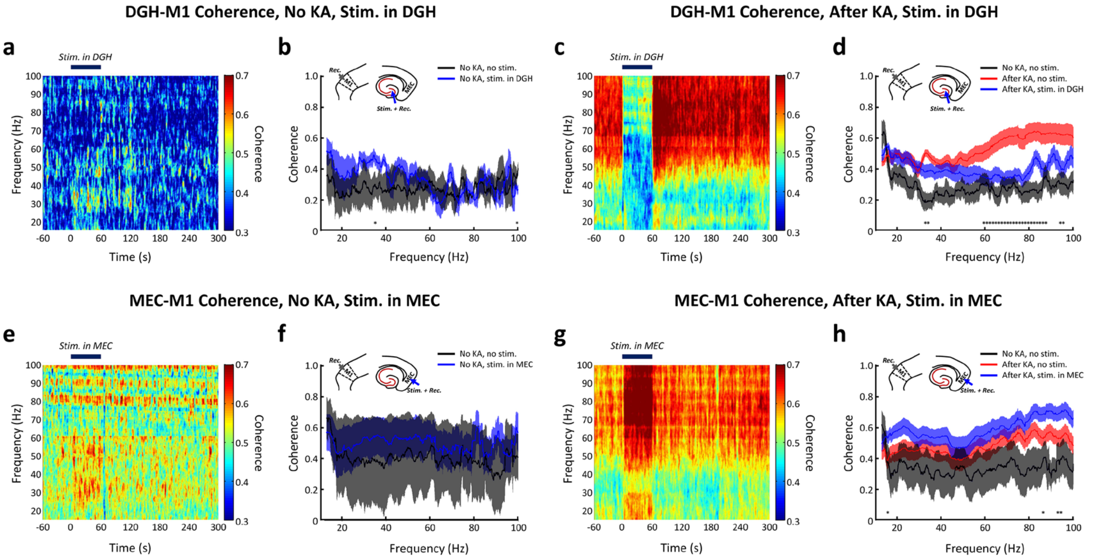

Supplementary Figure 15. Selectively activating DGH GABAergic interneurons decreases beta-gamma band LFP coherence. (**a-d**): LFP coherence spectrum (a, c) and mean coherence (b, d) between the DGH and M1 before (a-b; n = 3 mice) and after kainate administration (c-d; n = 6 mice). (**e-h**): LFP coherence spectrum (e, g) and mean coherence (f, h) between the MEC and M1 before (e-f; n = 3 mice) and after kainate (g-h; n = 6 mice). Light pulses were delivered at 130 Hz to the DGH (a-d) and MEC (e-h). Insets show the targeting sites for optical stimulation and electrical recording. Thick blue lines denote the 60 s stimulation periods. Error bars represent SEM. Stars represent significant differences ($p < 0.01$, paired t-test).

Supplementary Figure 16

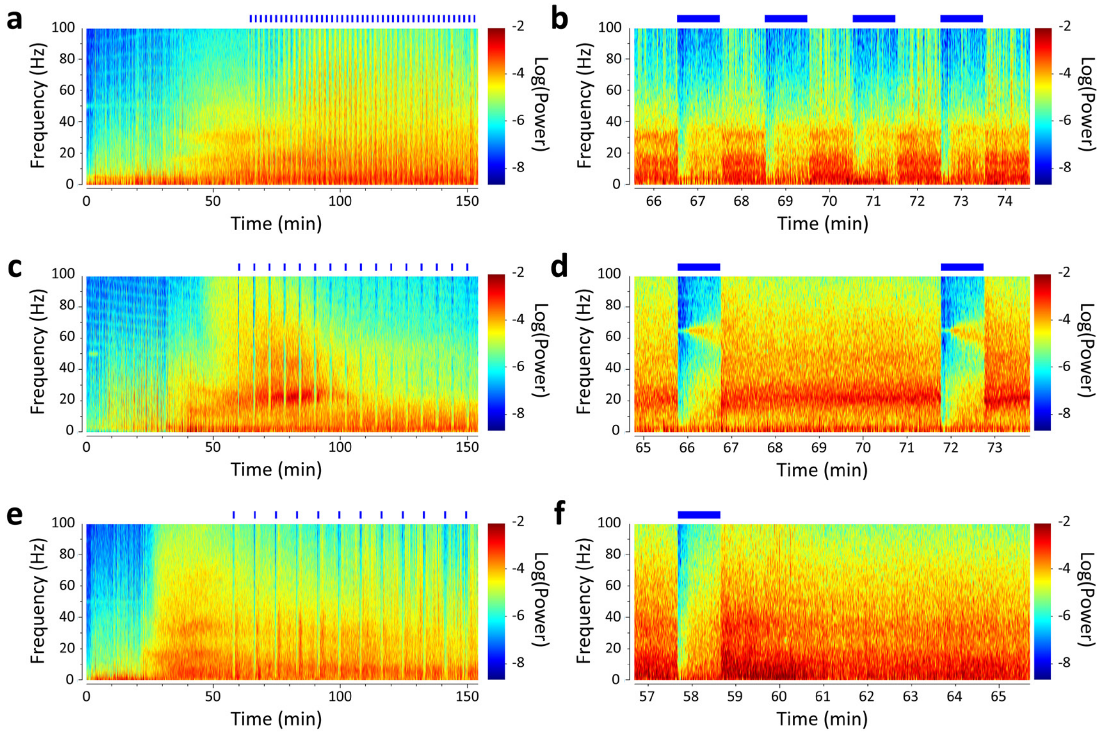

Supplementary Figure 16. Inhibitory effect on seizures using cyclic optogenetic stimulation with different inter-stimulation intervals. (**a, c, e**): Representative examples of spectrograms of LFPs in the DGH of intrahippocampal kainate mice with cyclic optogenetic stimulations. Light pulses (473 nm, 130 Hz, 5 ms pulse duration, 60 s on per stimulation cycle) were delivered to the DGH during a 90 min period. Data were normalized to the maximal $\log_{10}$(power) value across the entire 0–100 Hz frequency interval. Cyclic stimulation with short interstimulation interval (a) exhibited no significant effect on the progress of seizures, whereas cyclic stimulation with longer interstimulation intervals (c, e) showed a long-lasting inhibitory effect on seizures. (**b, d, f**): Enlarged views of stimulation periods with different interstimulation intervals. The interstimulation intervals were 60 s on and 60 s (a, b), 300 s (c, d), or 440 s (e, f) off, respectively. Light stimulation caused a significant and instant decrease in LFP activity, especially in the theta-gamma band. Each thick blue bar represents a 60 s light stimulation period.

Supplementary Figure 17

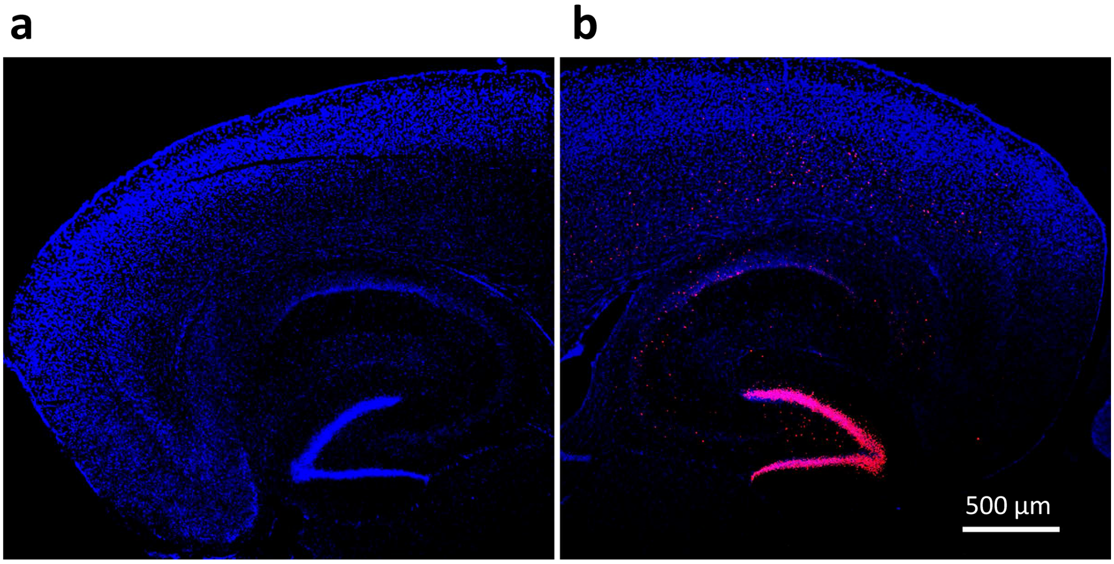

Supplementary Figure 17. CFos expression in hippocampal-entorhinal cortex (HP-EC) region before and after seizures. (**a**): Colabeling of cFos (red) and DAPI (blue) prior to intrahippocampal kainate. Virtually no cFos labeled cells were observed in the hippocampus and MEC of control VGAT-ChR2 mice. (**b**): Colabeling of cFos (red) and DAPI (blue) 2 h after KA injection. Strong cFos labeling was observed throughout most regions of the hippocampal formation, particularly in the DGH.
